# Structural and biological insights into *Klebsiella pneumoniae* surface polysaccharide degradation by a bacteriophage K1 lyase: implications for clinical use

**DOI:** 10.1186/s12929-022-00792-4

**Published:** 2022-02-07

**Authors:** I-Fan Tu, Tzu-Lung Lin, Feng-Ling Yang, I-Ming Lee, Wei-Lin Tu, Jiahn-Haur Liao, Tzu-Ping Ko, Wen-Jin Wu, Jia-Tsrong Jan, Meng-Ru Ho, Ching-Yi Chou, Andrew H.-J. Wang, Chung-Yi Wu, Jin-Town Wang, Kai-Fa Huang, Shih-Hsiung Wu

**Affiliations:** 1 Institute of Biological Chemistry, Academia Sinica, No. 128 Academia Road Section 2, Nan‑Kang, Taipei, 115 Taiwan; 2 Department of Microbiology, National Taiwan University Hospital, Taipei, 100 Taiwan; 3 Genomics Research Center, Academia Sinica, Taipei, 115 Taiwan; 4 Department of Chemistry, National Taiwan University, Taipei, 106 Taiwan

**Keywords:** *Klebsiella pnuemoniae*, K1 capsular polysaccharide, Tailspike protein, Polysaccharide lyase, Pyruvylation, Acetylation, β-Helix

## Abstract

**Background:**

K1 capsular polysaccharide (CPS)-associated *Klebsiella pneumoniae* is the primary cause of pyogenic liver abscesses (PLA) in Asia. Patients with PLA often have serious complications, ultimately leading to a mortality of ~ 5%. This K1 CPS has been reported as a promising target for development of glycoconjugate vaccines against *K. pneumoniae* infection. The pyruvylation and *O*-acetylation modifications on the K1 CPS are essential to the immune response induced by the CPS. To date, however, obtaining the fragments of K1 CPS that contain the pyruvylation and *O*-acetylation for generating glycoconjugate vaccines still remains a challenge.

**Methods:**

We analyzed the digested CPS products with NMR spectroscopy and mass spectrometry to reveal a bacteriophage-derived polysaccharide depolymerase specific to K1 CPS. The biochemical and biophysical properties of the enzyme were characterized and its crystal structures containing bound CPS products were determined. We also performed site-directed mutagenesis, enzyme kinetic analysis, phage absorption and infectivity studies, and treatment of the *K. pneumoniae*-infected mice with the wild-type and mutant enzymes.

**Results:**

We found a bacteriophage-derived polysaccharide lyase that depolymerizes the K1 CPS into fragments of 1–3 repeating trisaccharide units with the retention of the pyruvylation and *O*-acetylation, and thus the important antigenic determinants of intact K1 CPS. We also determined the 1.46-Å-resolution, product-bound crystal structure of the enzyme, revealing two distinct carbohydrate-binding sites in a trimeric β-helix architecture, which provide the first direct evidence for a second, non-catalytic, carbohydrate-binding site in bacteriophage-derived polysaccharide depolymerases. We demonstrate the tight interaction between the pyruvate moiety of K1 CPS and the enzyme in this second carbohydrate-binding site to be crucial to CPS depolymerization of the enzyme as well as phage absorption and infectivity. We also demonstrate that the enzyme is capable of protecting mice from K1 *K. pneumoniae* infection, even against a high challenge dose.

**Conclusions:**

Our results provide insights into how the enzyme recognizes and depolymerizes the K1 CPS, and demonstrate the potential use of the protein not only as a therapeutic agent against *K. pneumoniae*, but also as a tool to prepare structurally-defined oligosaccharides for the generation of glycoconjugate vaccines against infections caused by this organism.

**Supplementary Information:**

The online version contains supplementary material available at 10.1186/s12929-022-00792-4.

## Background

For more than two decades, pathogenic *Klebsiella pneumoniae* has been a community-acquired infection causing pyogenic liver abscesses (PLAs) in Asia. Patients with PLA often have serious complications, such as endophthalmitis, meningitis, and necrotizing fasciitis, ultimately leading to a mortality of ~ 5% [1]. Survivors of PLA-associated meningitis and endophthalmitis usually suffer from either severe neurological sequelae or blindness, respectively. Previous studies indicated that nearly all PLA *K. pneumoniae* strains possess a hypermucovisous phenotype attributed to their unique capsular polysaccharides (CPSs) [2], particularly the K1 CPS [3]. As an alternative to treatment with antibiotics, monoclonal antibodies against the K1 CPS of *K. pneumoniae* can enhance phagocytosis to combat invasion of the bacterium and protected mice from *K. pneumoniae*-induced liver abscess and lethality [4], suggesting that the K1 CPS is a promising target for vaccine development against *K. pneumoniae* infection [5]. On the basis of previous studies, however, whole CPS isolates from pathogenic bacteria could only induce relatively poor and T cell-independent immune responses [6]. In contrast, glycoconjugate vaccines generated by conjugating CPS to a carrier protein can elicit a stronger and long-lasting immune response [7–9].

The chemical structure of the K1 CPS comprises repeating units of the trisaccharide (→ 3)-β-D-Glc*p*-(1 → 4)-[2,3-(*S*)-pyruvate]-β-D-Glc*p*A-(1 → 4)-α-L-Fuc*p*-(1 →). The pyruvylation of the glucuronic acid and *O*-acetylation of the fucose are essential to the immune response induced by the K1 CPS [10]. In an attempt to fragment K1 CPS into oligosaccharides via chemical methods, we failed to retain the pyruvylation and *O*-acetylation modifications in the products [10]. Recently, the development of a K1 CPS-containing glycoconjugate vaccine against K1 *K. pneumonia* infection was reported [11]. The polysaccharide was enzymatically produced in glycoengineered *Escherichia coli*, but a low degree of pyruvylation was observed. Thus, alternate ways of accessing these glycans are important.

Many bacteriophages depolymerize the CPS of their host bacteria through their tailspike proteins (TSPs) [12]. The digested products usually comprise oligosaccharides with some repeating units, which retain the immunogenicity of CPS [13, 14]. In earlier work, we isolated a bacteriophage (NTUH-K2044-K1-1) whose genome encodes a K1 CPS-specific depolymerase [15]. Administration of this phage or the recombinant CPS depolymerase provided significantly increased survival in mice infected with the K1 *K. pneumoniae* NTUH-K2044 or an extended-spectrum β-lactamase (ESBL)-producing K1 strain [15]. Further investigation demonstrated that the CPS depolymerase removed the capsule from *K. pneumoniae *in vivo, enabling the host immune system to kill the bacterium, implicating the therapeutic potential of the enzyme against *K. pneumonia* [16].

In the present study, we analyzed the digested products of K1 CPS with NMR spectroscopy and mass spectrometry to reveal that the NTUH-K2044-K1-1 CPS depolymerase is a K1 lyase. Crystal structures of the enzyme containing bound products revealed two distinct carbohydrate-binding sites with unusual features compared to other TSP depolymerases. Through site-directed mutagenesis, phage absorption and infectivity studies, and treatment of the *K. pneumoniae*-infected mice with the mutant enzymes, we have obtained deeper insights into how this enzyme recognizes and depolymerizes K1 CPS on the surface of *K. pneumoniae*.

## Materials and methods

### Protein production

The gene *orf34* (Genbank: BAP15746) of bacteriophage NTUH-K2044-K1-1, which encodes the K1 CPS-specific depolymerase K1 lyase (amino acids 1–651), was amplified and sequenced as described previously [15]. The *orf34* DNA was inserted into the vector pET28a (Novagen) via *Nde*I and *Xho*I cloning sites and the vector was transformed into the *E. coli* BL21 (DE3) (Novagen). The cells were grown in Luria- Bertani (LB) medium supplemented with 50 μg/mL kanamycin at 37 °C until the cell density reached OD600 of 0.4–0.6. The cultured cells were induced with 0.4 mM IPTG at 20 °C for 12–16 h. Then, the cells were harvested by centrifugation (6,000 rpm) at 4 °C for 30 min and suspended in buffer A (25 mM Tris–HCl and 100 mM NaCl, pH 7.5). The cells were lysed by passing through a French Press (Constant System Ltd, Constant System TS 2.2kw) three times and the lysate was clarified by centrifugation (9000 rpm) at 4 °C for 30 min. The supernatant was loaded onto an open column filled with nickel-chelating Sepharose resin (GE Healthcare) pre-equilibrated with buffer A. The column was washed with buffer B (25 mM Tris–HCl, 100 mM NaCl and 90 mM imidazole, pH 7.5) and the recombinant protein was eluted with buffer C (25 mM Tris–HCl, 100 mM NaCl and 200 mM imidazole, pH 7.5). The eluted fractions were pooled and then dialyzed against buffer D [25 mM Tris–HCl, 100 mM NaCl and 10% (v/v) glycerol, pH 7.5] at 4 °C for 12 h. The recombinant protein was further purified by a Superdex-200 gel-filtration column (GE-Healthcare), leading to near homogeneity. Site-directed mutagenesis experiments were conducted by using the QuikChange II site-directed mutagenesis Kit (Stratagene) and following the manufacturer's instruction. Protein concentration was measured spectrophotometrically on the basis of the extinction coefficient 84,230 cm^−1^ M^−1^ calculated from the protein sequence [17].

### Agar overlay assay

Agar overlay assay was performed according to a previous protocol [18]. Briefly, LB agar in a petri dish was overlaid with 5 mL of top agar, which was pre-incubated with fresh culture of *K. pneumoniae* NTUH-K2044 and an adequate amount of phage. After an overnight incubation at 37 °C, the infectivity of phage was evaluated by measuring the dimension of clear zone plus surrounded translucent halo on the surface of agar.

### Extraction of K1 CPS

We used the *K. pneumoniae* strain NTUH-K2044 ∆*wbbO* to extract the K1 CPS, because this strain lacks the O-antigen in lipopolysaccharide and thus avoids the contamination of lipopolysaccharide The protocol of extraction was referring to a previous report [10]. Briefly, the bacteria were collected from an overnight culture on solid agar and suspended in distilled water. The suspension was heated at 100 °C for 10 min and then cooled down to room temperature. The bacterial debris was removed by centrifugation at 15000xg for 20 min. The polysaccharide in the supernatant was precipitated by adding ice-cold acetone, and crude extract of K1 CPS was obtained by centrifugation at 12,000×*g* for 20 min. The crude extract was incubated with ribonuclease (Roche) and deoxyribonuclease I (Roche) at 37 °C for 24 h, and added with protease K in 10 mM Tris–HCl, pH 7.4, and then further incubated for 6–8 h. After heated at 100 °C for 10 min and clarified by centrifugation, the supernatant was dialyzed extensively against water using an 8–10 kDa cutoff membrane and lyophilized. Prior to mass spectrometry analysis and enzymatic assays, the K1 CPS was further purified by a TSK HW-65F gel-filtration column.

### NMR sample preparation and data collection

Seven mg of the K1 lyase-digested products was dissolved in 300 μl D_2_O containing 33 μM DSS as an internal chemical shift standard, with the pH value of the sample being 4.65. The 2D DQF-COSY spectrum of the products was collected at 298 K on a Bruker 800 MHz NMR spectrometer equipped with a cryogenic probe. All experiments were carried out with the standard pulse sequences provided by Bruker software and the responsive data were processed with Bruker TopSpin program. The data acquisition parameters are as follow: 2048 (F2) by 512 (F1, increments) data matrix, 56 scans for each increment, 9 ppm spectral width in both dimensions. Data processing parameters are as follow: 4096 (F2) by 1024 (F1) data matrix. A 60-degree shifted sinebell window function was applied in both dimensions.

### Dot blot analysis

Antiserum raised against the K1 CPS was obtained from the Laboratory of HealthCare Associated Infection, Health Protection Agency [19]. Column-purified K1 CPS and its K1 lyase-digested products with quantities in the range of 0.1 to 500 μg were loaded onto a PVDF membrane (0.45 μm, Immobilon-P, Millipore) by using a vacuum-driven slot blot filtration manifold (Hoefer). The membrane was blocked with 5% non-fat milk dissolved in 1X PBS buffer at room temperature for 1 h and then incubated with the antiserum (1/:10,000 dilution by 0.1% Tween 20 in 1 × PBS) for 1 h. Subsequently, the membrane was incubated with a secondary antibody (1:20,000 dilution by 0.1% Tween 20 in 1 × PBS) for 1 h and the chemiluminescence-based immunodetection was performed by using the substrate ECL (Millipore).

### Mass spectrometry analysis

Mass spectrometry analyses were performed at the Mass Core Facility of Genomics Research Center, Academia Sinica (Taipei, Taiwan). LC–ESI–MS and LC–ESI–MS-MS analyses were done on a LTQ Orbitrap XL ETD mass spectrometer (Thermo Fisher Scientific, San Jose, CA) equipped with standard ESI ion source. 5 μL of sample in 80% ACN/H_2_O and 0.1% FA was injected at a flow rate of 50 μL/min by Ultimate 3000 RSLC system (Dionex Corporation, Sunnyvale, CA). The conditions for full-scan MS are as follows: mass range m/z 100–2000 and resolution 60,000 at m/z 400. The target ions were sequentially isolated for MS2 by LTQ. Electrospray voltage was maintained at 4 kV and capillary temperature was set at 275 °C.

### Thermal stability analysis

The thermal stability of recombinant enzymes was evaluated by monitoring the change of circular dichroism (CD) spectra under various temperatures. The CD experiments were performed on a J815 spectrometer (Jasco) using a 10 mm-path quartz cuvette. The recombinant enzyme at the concentration of 3 μM in 30 mM sodium phosphate and 100 mM sodium fluoride, pH 7.5, was gradually heated from 25 to 95 °C at a speed of 1 °C /min, and the CD spectrum between 260 and 200 nm at each temperature was recorded at a scan speed of 20 nm/min and a data pitch of 0.5 nm. The obtained spectra were further smoothed and normalized by using the software *Spectra Manager* (Jasco). The melting point temperature was estimated by measuring the change of mean residue ellipticity at 216 nm for the gradually elevated temperatures.

### Analytical ultracentrifugation analysis

Ultracentrifugation sedimentation experiments were performed at 20 °C and at 20,000 rpm in a Beckman XL-A analytical ultracentrifuge (Beckman Instruments, Fullerton, Calif, USA) equipped with the standard double-sector centerpieces and using an An-60 Ti rotor. The UV absorption at 280 nm was scanned every 4 min for 250 scans. The data were analyzed with the software SEDFIT. The protein sample was in a buffer of 25 mM Tris–HCl and 100 mM NaCl, pH 7.5. The sample was visually checked for clarity after ultracentrifugation. No precipitation was observed.

### Enzyme activity assay

The enzymatic activity of K1 lyase on the substrate K1 CPS was evaluated by monitoring the elevated UV absorption at 232 nm, since the degraded products contained unsaturated oligosaccharides [12]. The assay was performed at 25 °C in an Ultrospec 4000 UV/Visible spectrophotometer (Amersham Pharmacia Biotech, Piscataway, NJ, USA) using an 1 ml cuvette. The reaction was started by adding the recombinant enzyme (10 μg/ml) to the mixture containing 40 μg/ml K1 CPS and 3 mM MgCl_2_ in 25 mM Tris–HCl, pH 7.5. The increase in UV_232_ absorption was monitored for 15 min. Since the CPS extracts were heterogeneous, the enzymatic activity was represented as ∆A_232_‧min^−1^‧mg^−1^.

The enzyme kinetic study of K1 lyase and its mutant was conducted by measuring the production of reducing sugars using 3-methyl-2-benzothiazolinehydrazone (MBTH) [20]. A 20-μl reaction mixture containing 10 μg/ml enzyme and K1 CPS at varying concentrations (0–6.5 mg/ml) was incubated at 25 °C for 3 min and then added with 0.5 N NaOH to stop the reaction. Subsequently, the reaction mixture was added with dithiotreitol/MBTH solution (1:3 mg/ml ratio, Sigma-aldrich) and then heated at 80 °C for 15 min. After heating, the mixture was treated with the solution containing 0.5% ammonium iron(III) sulfate, 0.5% sulfamic acid and 0.25 N HCl, and then cooling down to room temperature. The initial velocity of the reactions was determined by monitoring the change of A620 using a Tecan Infinite M1000 pro (*Tecan*, Männedorf, Switzerland). Since possible product inhibition was observed for wild-type K1 lyase, the equation *v*_*0*_ = *V*_max_ [S]/(*K*_m_ + [S] + [S]^5^/*K*_i_^4^) was used to fit the initial velocity (*v*_*0*_) data by nonlinear regression with the software *KaleidaGraph* (Synergy Software, Reading, Pennsylvania, USA), where *V*_max_ is the limiting rate, S the substrate concentration, *K*_m_ the Michaelis constant, and *K*_i_ the inhibition constant [21]. Because no significant product inhibition was observed for the R472A mutant, the equation *v*_*0*_ = *V*_max_ [S]/(*K*_m_ + [S]) was used to fit the initial velocity data to obtain the *K*_m_ and *V*_max_ values. The *k*_cat_ values were calculated by *V*_max_ dividing to enzyme concentration. Correlation coefficients of better than 0.955 were obtained throughout the fittings.

### Crystallization and X-ray data collection

Purified K1 lyase was concentrated to ~ 15 mg/mL and subjected to crystallization screening using ~ 1,000 conditions at the Protein X-ray Crystallography Facility, Institute of Biological Chemistry, Academia Sinica (Taipei, Taiwan). The resulting hit conditions were further refined manually. Finally, two crystallization conditions were selected: (i) 15% (w/v) PEG4000, 0.2 M imidazole malate, pH 6.0, and (ii) 15% (w/v) PEG6000, 0.1 M citrate (pH 5.5), and 0.019 M n-decyl-*N*,*N*-dimethylglycine. The crystals were grown at 20 °C by mixing the protein solution with equal volume of crystallization buffers via the sitting-drop vapor-diffusion method. For condition (i), the rod-like crystals with dimensions reaching 0.15 × 0.10 × 0.10 mm appeared within 3 days; for condition (ii), plate-shaped crystals with dimensions reaching 0.25 × 0.20 × 0.05 mm appeared in one day. Since the crystals grown in condition (i) diffracted X-ray stronger, we used the crystals to solve the phase problem and to carry out the soaking experiments with K1 CPS. A multiple wavelength anomalous dispersion (MAD) data at 1.95-Å resolution was collected at the beamline 44XU of SPring-8 (Hyogo, Japan), using Pt-labeled crystals obtained by soaking the crystals in 2 mM K_2_Pt(CNS)_6_ at 20 °C for 3 days. The high-resolution X-ray data were collected at the beamline 13B1 of National Synchrotron Radiation Research Center (NSRRC) (Hsinchu, Taiwan) and the Molecular Biology Consortium beamline of Advanced Light Source (Berkeley, USA). For structure of product-bound K1 lyase, the crystals were soaked in crude extract of *K. pneumoniae* K1 CPS (0.5 mg/mL) at 20 °C for 24 h, and the X-ray data were collected at the NSRRC beamlines 15A1, 13B1, or 13C1, and the SPring-8 beamline 12B2. Before mounting on the goniometer, the crystals were briefly immersed in reservoir solution containing 10% (v/v) glycerol as cryoprotectant. All diffraction data were processed and scaled with the *HKL-2000* package [22]. The data collection statistics are listed in Table 1. The space group for crystals grown in condition (i) and (ii) is *P*1 and *C*2, respectively, with asymmetric unit comprising 2 and 4 K1 lyase trimers. The cryoprotectant for crystals of condition (ii) was 15% (v/v) glycerol.

**Table 1:** Data collection and refinement statistics

	Pt derivative		Native	Native	Product-bound
	High energy remote	Inflection	P1 crystal form	C2 crystal form	P1 crystal form
Data collection					
Wavelength (Å)	1.05372	1.07194	1.0	1.0	1.0
Space group	*P*1	*P*1	*P*1	*C*2	*P*1
Resolution (Å)	30–1.95	30–1.95	30–1.48	30–2.78	30–1.46
Unit cell dimensions					
*a*, *b*, *c* (Å)	90.77, 100.83, 125.38	90.79, 100.84, 125.40	90.28, 100.57, 125.03	363.95, 239.63, 121.63	90.63, 100.99, 125.38
*α, β*, *δ* (°)	80.50, 70.34, 83.74	80.50, 70.34, 83.74	80.50, 70.32, 83.43	90, 100.13, 90	80.40, 70.48, 83.99
Total observations	1,733,160	1,720,628	2,458,178	834,222	1,333,537
Unique reflections	291,399	291,203	656,693	257,393	673,887
Multiplicity	5.9 (5.9)	5.9 (5.9)	3.7 (3.7)	3.2 (3.2)	2.0 (2.0)
Completeness (%)	97.7 (96.3)	97.7 (96.2)	97.0 (95.4)	99.7 (99.9)	94.4 (92.9)
*I*/σ(*I*)	21.96 (4.81)	21.27 (4.30)	25.44 (2.28)	11.24 (2.47)	19.84 (2.14)
*R*_merge_ (%)	15.0 (76.8)	15.3 (82.6)	7.3 (74.1)	13.9 (62.5)	3.2 (24.3)
Refinement					
Resolution (Å)			30.0–1.48	29.95–2.78	23.27–1.46
Reflections [> 0σ(*F*)], working/test			590,498/32,922	228,380/12,744	606,381/33,731
*R* factor/*R*_free_			0.137/0.184	0.182/0.219	0.114/0.148
R.m.s.d., bond lengths (Å)/ angles (°)			0.018/1.697	0.017/1.851	0.009/1.514
Average *B* factor (Å^2^)/No. of atoms					
Protein			21.1/28,967	29.9/58385	16.7/29,026
Sugar and other ligands			35.0/286	58.3/220	27.0/534
Water			36.8/4,043	26.3/1344	33.4/4914
Ramachandran plot, residues in (%)					
Most favored regions			89.1	84.3	89.2
Additionally allowed regions			10.1	14.7	10.0
Generously allowed regions			0.8	1.0	0.8
Disallowed regions			0.0	0.0	0.0
PDB code			7W1C	7W1D	7W1E

Values in parentheses correspond to the highest resolution shell

### Structure determination and refinement

The crystal structure of K lyase was solved by the MAD-phasing method using the X-ray data collected at the wavelengths of inflection and high-energy remote of Pt. The positions of 23 Pt atoms in the asymmetry unit, with occupancies between 0.1 and 1.0, were determined with *Shelx C/D* and refined with *BP3* [23, 24]. The initial phases were improved by density modification with *Solomon* [25]. Approximate 84% model was then automatically traced into the electron density map with *Buccaneer* [26], and the remainder was manually built with *Coot* [27]. The resulting model was subjected to computational refinement with the program *REFMAC5* [28]. Throughout refinement, a randomly selected 5% of the data was set aside as a free data set, and the model was refined against the remaining data with *F* > 0 as a working data set. Subsequently, iterative rounds of model adjustment with *Coot* and refinement with *REFMAC5* were performed using the 1.48-Å resolution data set to improve the quality and completeness of the structure. The well-ordered water molecules were located with *Coot*. Finally, the refinement converged at a final R factor and R_free_ of 0.137 and 0.184, respectively. The stereochemical quality of the refined structure was checked with the program *PROCHECK* [29]. The final refinement statistics are listed in Table 1. Regarding the structure of product-bound form, the initial difference Fourier map was obtained by using the refined structure. For the structure of *C*2 crystal form, the initial phase was obtained by the molecular replacement phasing method with *Molrep* and using a K1 lyase trimer as the search model [30]. The subsequent model building and refinements were the same as described above. The molecular figures were generated with *PyMOL* (Schrödinger, New York, USA).

### Generation of mutant phages

The protocol of site-directed mutagenesis to the genome of phage NTUH-K2044-K1-1 was referring to a previous study with some modifications [31]. A trimethoprim resistant gene (DHFR) was cloned into an *Apa*L1 site of the plasmid pKD46 carrying phage λ Red recombinase under an inducible arabinose promoter which can promote the recombination [32, 33]. The resulted DHFR-pKD46 was transformed into the *K. pneumoniae* strain NTUH-K2044 via electroporation. The plasmids orf34 R378A::pET28c and orf34 R472A::pET28c, serving as the substrates of recombination, were transformed into the NTUH-K2044/DHFR-pKD46 cell.

The NTUH-K2044/DHFR-pKD46 cell carrying the plasmid orf34 R378A::pET28c or orf34 R472A::pET28c was induced by 1 mM arabinose for 3 h and then infected with phage NTUH-K2044-K1-1 at a titer of 100 pfu (plaque forming unit) via the agar overlay method. Among the resulting single plaques of phage, the mutant phages were selected by sequencing the orf34 gene. The homogenous preparation of mutant phages was achieved by three rounds of re-plating and orf34 gene sequencing.

### Phage absorption assay

The *K. pneumoniae* strain NTUH-K2044 was grown in LB broth until OD_600_ reaching ~ 1.0. The bacterial culture was incubated with an equal volume of phage (10^5^ pfu/ml) at 37 °C for 5 min. Subsequently, the culture was filtered (0.22 μm) and the free phages in the filtrate were counted by using the agar overlay method. The reduction in titer of free phages reflects the number of phages adsorbed on the surface of bacterial host.

### Animal study

The protocol of animal study was referring to a previous report with some modification [15]. Three groups of mice, each containing five 5-week-old female BALB/cByl mice, were inoculated intraperitoneally with 8.0 × 10^7^ colony-forming units (CFUs) of *K. pneumoniae* strain NTUH-K2044. After 30 min of *K. pneumoniae* infection, the mice were administrated intraperitoneally with 25 µg of the wild-type or mutant K1 lyase. The other two groups of mice were inoculated with NTHU-K2044 alone or phosphate-buffered saline as the negative and positive control, respectively. The mortality of mice was recorded for 8 days. Survival rate was analyzed by Kaplan–Meier method with a log-rank test; the difference was considered statistically significant at *P* < 0.05.

To evaluate the toxicity of K1 lyase itself in mice, 4 mice were administrated intraperitoneally with K1 lyase at the dose of 100 μg/per mouse. The change in behavior and body weight of the mice was monitored daily for 8 days. A mouse received phosphate-buffered saline alone served as the control.

## Results

### K1 lyase functions as a trimer and turns out repeating units of trisaccharide

**Fig. 1:**
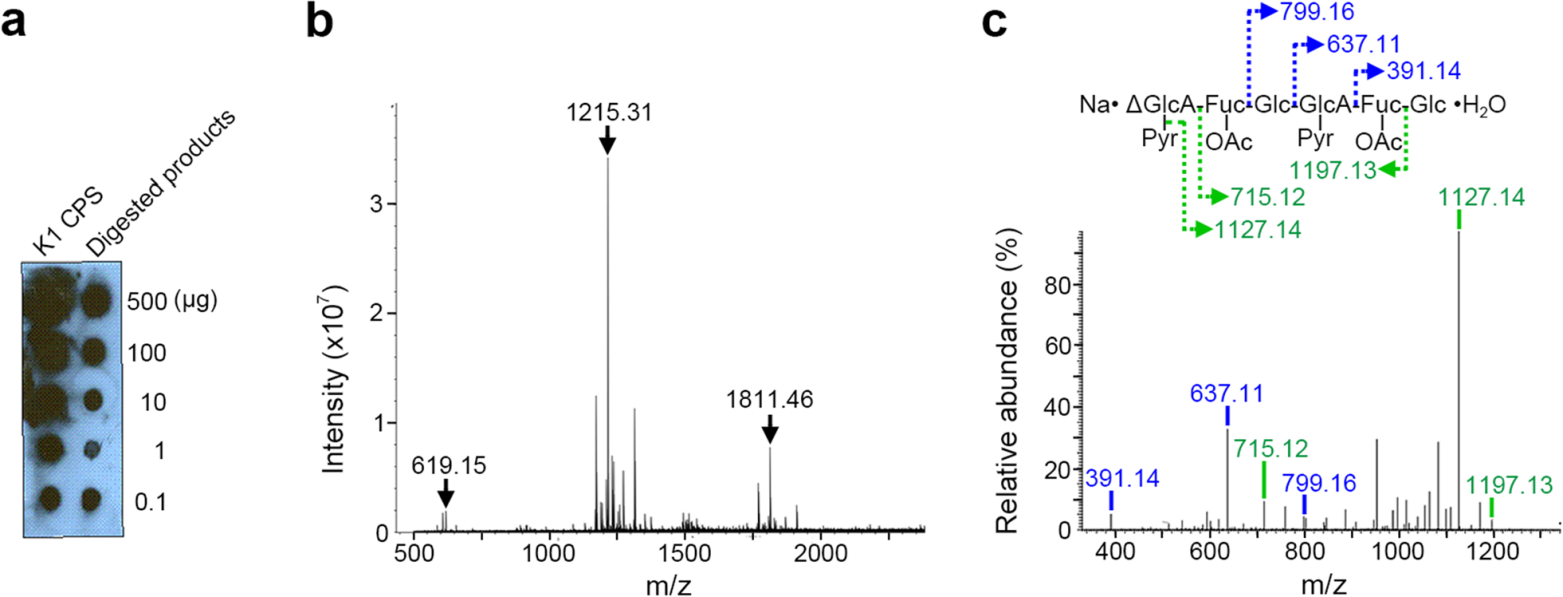
Analysis of the K1 lyase-degraded products of K1 CPS. **a** Dot blot analysis of the K1 CPS with or without K1 lyase treatment using anti-serum generated from the K1 CPS. **b** LC–ESI–MS analysis of the K1 lyase degraded products of the K1 CPS. **c** LC–ESI–MS-MS analysis of the main digested product (m/z = 1215.31) of K1 CPS. *Pyr* pyruvate; *OAc* acetyl group the C-2 or C-3 hydroxyl groups

Recombinant K1 CPS depolymerase with a His-tag was overexpressed in *E. coli* and purified as a homotrimeric protein (Additional file 1: Fig. S1a). The purified enzyme generated a translucent halo when spotted on agar inoculated with K1 *K. pneumoniae* NTUH-K2044 (Additional file 1: Fig. S1b), manifesting its CPS depolymerization activity. The products showed a UV absorption at 232 nm (Additional file 1: Fig. S1c), a double bond signal at 6.1 ppm in the 1D-^1^H NMR spectrum, and the disappearance of the signal for the C-5 proton of the GlcA residue in the 2D-^1^H,^1^H DQF-COSY spectrum (Additional file 1: Fig. S1d), indicating that the enzyme is a polysaccharide lyase [34]. Antiserum generated from the K1 CPS recognized the digested products (Fig. 1a) showing they retained the antigenicity of the intact CPS. LC–ESI–MS analyses of the degraded products revealed a major oligosaccharide (m/z = 1215.31) consisting of two trisaccharide repeating units, accompanied by two minor products (m/z = 1811.46 and 619.15) corresponding to oligosaccharides of one and three repeating units (Fig. 1b). Notably, all of these products retained the pyruvylation and *O*-acetylation (Fig. 1c, Additional file 1: S1e). Further characterization of the enzyme revealed a relatively high thermal stability (*T*_m_ ~ 82 °C) and a maximal catalytic activity at 55 °C (Additional file 1: Fig. S1f, g). Treating the enzyme with 1% SDS led to nearly complete dissociation of the trimer into monomers, but ~ 20% enzymatic activity was retained (Additional file 1: Fig. S1h, i), suggesting that the trimeric structure is crucial for full CPS depolymerase activity, but that the monomer sufficiently catalyzes the lyase reaction.

### The overall structure of K1 lyase features a central β-helix for receptor binding

**Fig. 2:**
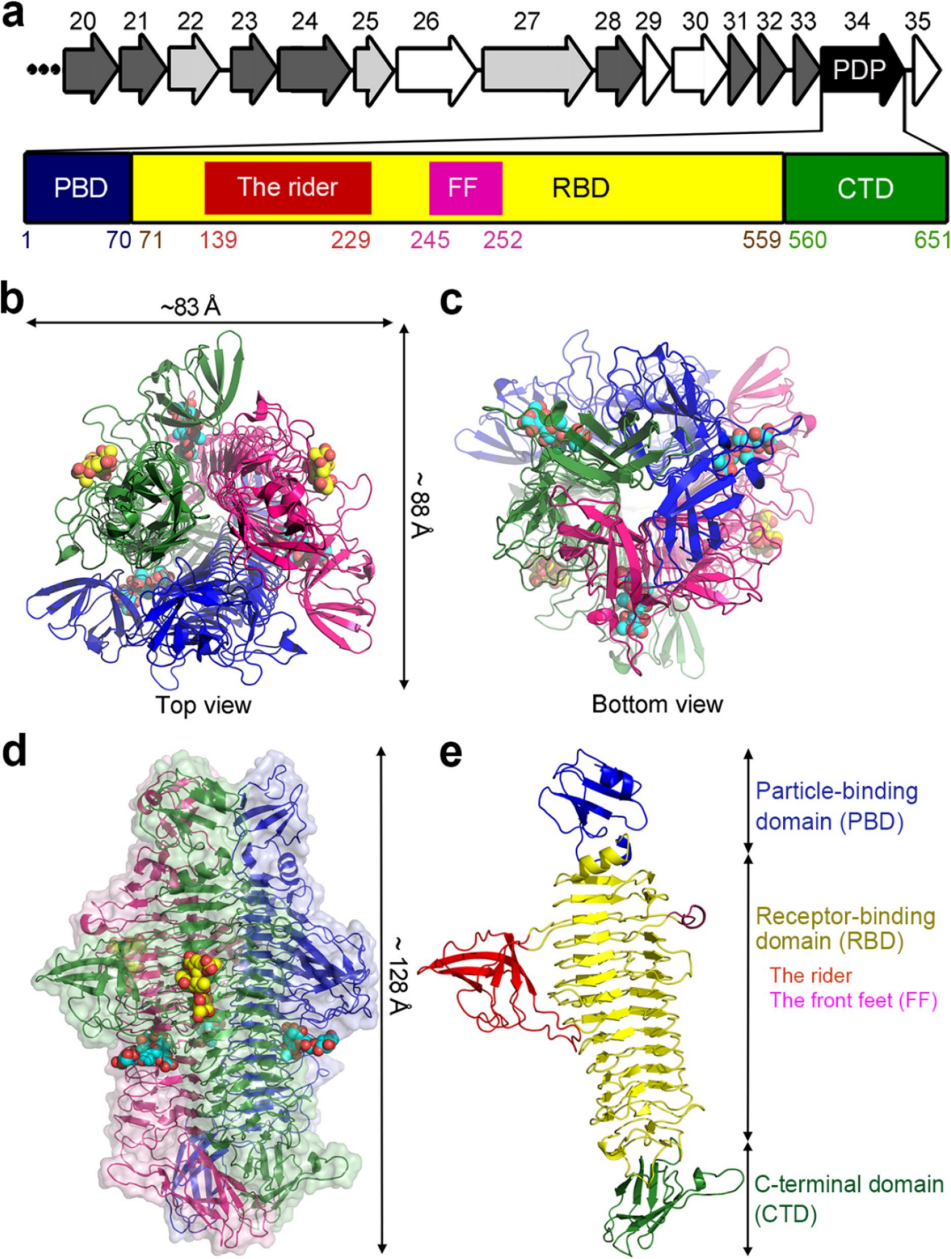
Overall structure of the K1 lyase. **a** The gene encoding a polysaccharide depolymerase (PDP) in the genome of bacteriophage NTUH-K2044-K1-1. The arrows represent the open reading frames 20–35 of the genome [15]. The polysaccharide depolymerase contains distinct domains, as described below, with the amino acid residues in the domains being further indicated. **b**, **c** and **d** The trimeric structure of the enzyme drawn as three-colored ribbons in top, bottom and side view, respectively. The bound trisaccharides at the solvent-accessible groove of individual subunits and the inter-subunit pockets are shown as yellow and cyan sphere models, respectively. The view in **d** is perpendicular to the trimer axis onto a solvent-accessible groove with the N-terminal end on top. A transparent surface is also shown. **e** Structure of individual subunit of K1 lyase. The domains are further indicated with colors as in **a**

The purified K1 lyase crystallized in the *P*1 and *C*2 space groups. The structure was solved by the Pt-MAD phasing method (Table 1). The *P*1 crystal contains two trimers of the enzyme in an asymmetric unit, and the *C*2 crystal has four (Additional file 1: Fig. S2a, b). Detailed structural analyses are mainly based on the *P*1 form because of its much higher resolution. The structures revealed a compact, rod-like, trimeric molecule with an overall length of ~ 128 Å and a diameter of ~ 86 Å at its widest part (Fig. 2a–d). Only limited structural differences between the two crystal forms were observed, with an average root-mean-square (rms) deviation of ~ 0.25 Å between all Cα atoms, indicating a rigid architecture of the enzyme. The three subunits are packed side-by-side to form a parallel, left-handed superhelix. As analyzed by PDBePISA (http://www.ebi.ac.uk/pdbe/pisa), ~ 30% (7565 out of 24,958 Å^2^) of the solvent-accessible surface in each subunit is buried, excluding a total surface area of 22,693 Å^2^ upon trimer formation. The shape of the monomer is reminiscent of a rider on a running horse (Fig. 2e). The body of the horse corresponds to the right-handed β-helix, from which two protruding structures constitute its “front feet” and the “rider”. The putative N-terminal particle-binding domain makes up the “head”, the C-terminal β-sandwich contains the “tail” and the “back feet” of the horse.

**Fig. 3:**
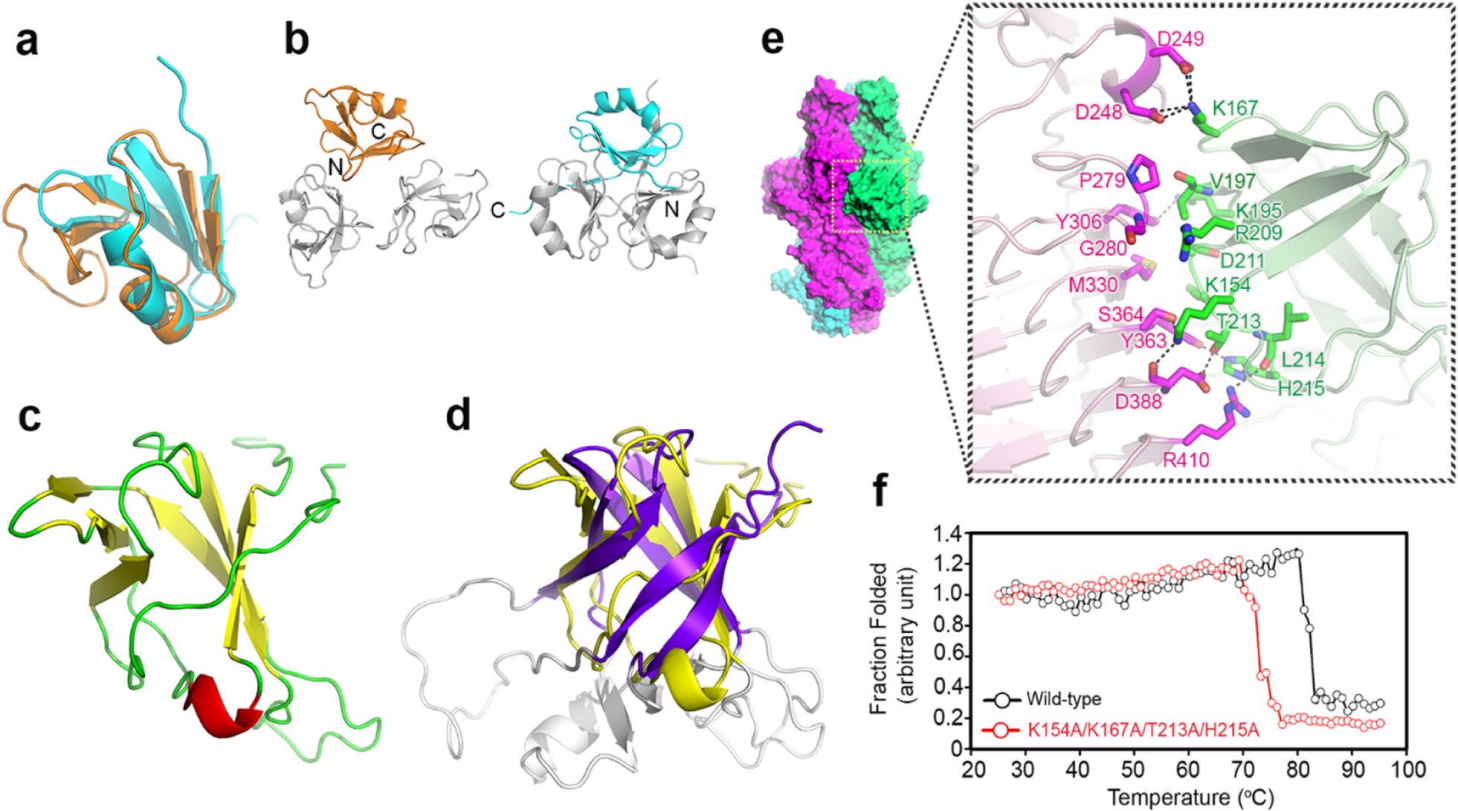
The N-terminal domain and the “rider” domain of K1 lyase. **a** Superimposition in the monomer of the N-terminal domain of the K1 lyase (orange) and the human collagen XVIII trimerization domain (cyan) [35]. **b** Comparison in the trimer of the two structures described in (**a**). The N-terminal and C-terminal end in a subunit of both structures are indicated. **c** The “rider” domain, a strikingly protruding structure at the central β-helix, displaying a β-barrel-like fold. **d** Superimposition of the “rider” domain (yellow) and the I-domain of bacteriophage P22 coat protein (purple) [36]. The unstructured loops in both structures are colored gray. **e** Interaction of the “rider” domain (green) with the β-helix of a neighboring subunit (magenta). **f** Comparison of thermal stabilities of wild-type (black) and mutant (red) K1 lyase, as evaluated by circular dichroism spectroscopy

The N-terminal domain (Glu^10^–Pro^70^) of each monomer folds into a barrel-like structure with a five-stranded β-sheet and a six-residue α-helix (Fig. 3a), stabilized by three buried hydrogen bonds (H-bonds) in addition to hydrophobic interactions. A search with the DALI server (http://ekhidna.biocenter.helsinki.fi/dali_server) revealed structural similarity to human collagen XVIII trimerization domain (by a rms deviation of 1.34 Å between the Cα atoms) (Fig. 3a, b) [35]. This fold is unusual among bacteriophage TSPs (Additional file 1: Fig. S3). In a trimer, three equivalent β-strands are laterally associated into a triangular prism but the α-helices are solvent exposed (Fig. 2b, left panel of 3b). Although the trimeric association is similar to that of human collagen XVIII, the N-terminal domain of the K1 lyase has fewer stabilizing interactions. Compared to the *P*1 structure, a slight rearrangement occurred in the *C*2 crystal (Additional file 1: Fig. S2c), indicating the flexible nature of the N-terminal domain.

**Fig. 4:**
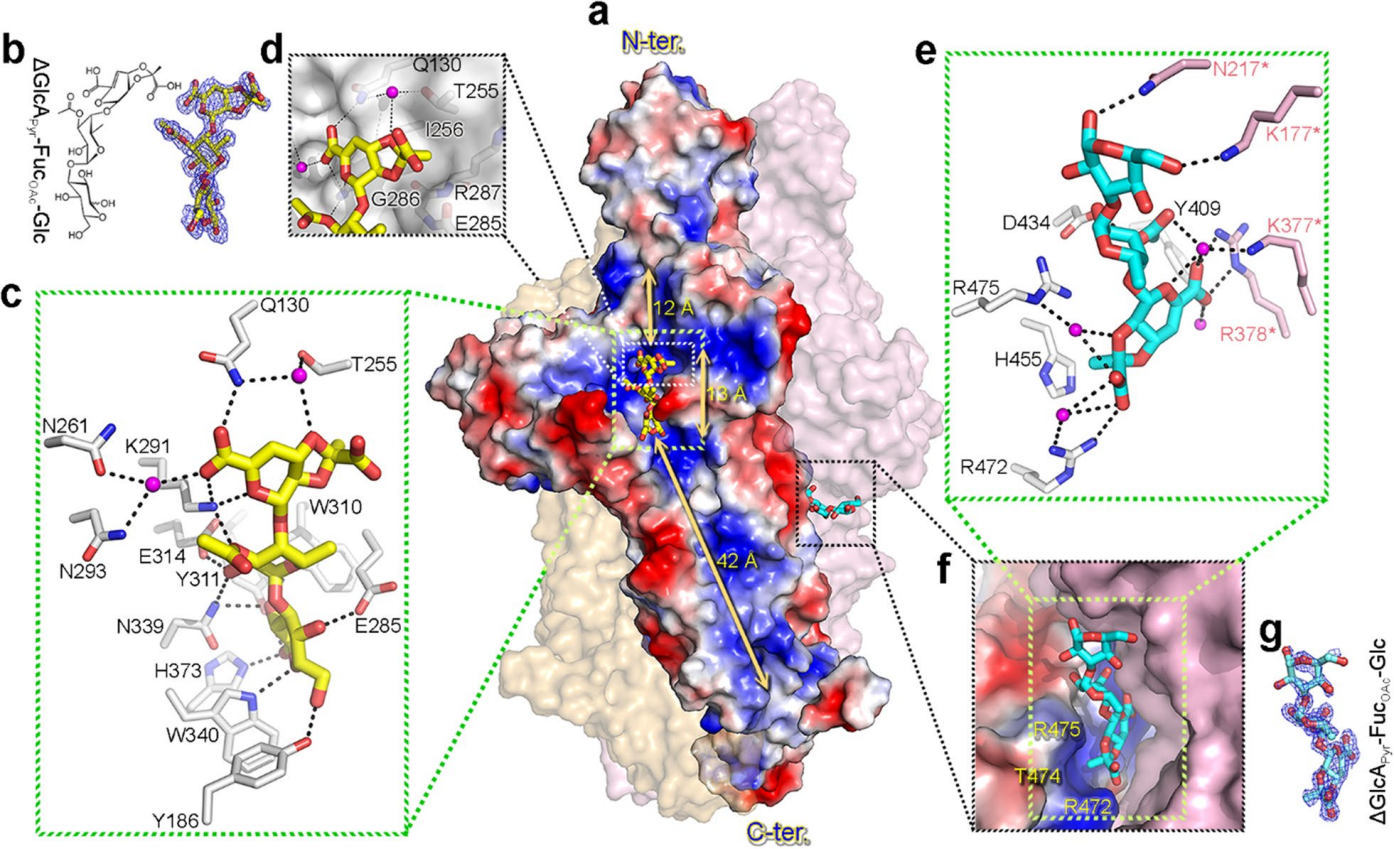
Two distinct carbohydrate-binding sites with bound trisaccharides. **a** Surface representation of the structure of trisaccharide-bound K1 lyase, with a view the same as in Fig. 2d. The surface charge potentials of the subunit are shown, with blue and red color representing the positive and negative charge, respectively. The lengths for different regions of the solvent-accessible groove are indicated. **b** The chemical structure and final refined model of the bound trisaccharide at the solvent-accessible groove of a subunit. The 1σ 2*F*_o_–*F*_c_ omit map around the refined model is also shown. Abbreviations: ΔGlcA_Pyr_ = [2,3-(*S*)-pyruvate]-β-D-∆4,5-Glc*p*A; Fuc_OAc_ = *O*-acetyl-α-L-Fuc*p*. **c** Detailed interaction of the bound trisaccharide with the enzyme at the solvent-accessible groove. **d** The pyruvyl group of the bound trisaccharide is docked to a small pocket in the solvent-accessible groove. **e** Detailed interaction of the bound trisaccharide with the enzyme at the inter-subunit pocket. **f** The pyruvyl group is docked to a deep pocket created by residues Arg^472^, Thr^474^ and Arg^475^. **g** The 1σ 2*F*_o_–*F*_c_ omit map around the refined model of a bound trisaccharide at the inter-subunit pocket

The central receptor-binding domain (Ala^71^–Asp^559^) of each monomer folds into a right-handed parallel β-helix consisting of 14 complete rungs, plus an extended α, β-mixed turn on the top, where an eight-residue α-helix covers the lumen of the β-helix, resulting in a well-formed hydrophobic core (Fig. 2e). The interior features the inward-facing stacks of β-branched or aromatic side chains (Additional file 1: Fig. S4a). The main body of the β-helix displays a kidney-shaped cross section with every rung being organized into three strands, B1, B2 and B3, separated by turns T1, T2 and T3 (Fig. 2e, Additional file 1: Fig. S4b), as seen in other TSPs (Additional file 1: Fig. S3). The parallel strands of B1, B2 and B3, respectively, merge into β-sheet b1, b2 and b3 and form the three faces of the β-helix. Notably, the β-sheet b1 and turns T1 and T3 define an elongated solvent-accessible groove (Fig. 4, Additional file 1: Fig. S4b), where the CPS depolymerization takes place, as described below.

Succeeding the β-helix, the polypeptide chain (Asp^560^–Leu^651^) loops out with respect to the subunit longitudinal axis and is organized into a β-sandwich consisting of a four-stranded and a three-stranded antiparallel β-sheets plus a four-residue 3_10_-helix (Fig. 2e). The β-sandwich is stabilized by hydrophobic interactions in the interior and H-bonds between loops connecting the β-strands. Furthermore, a protruding loop forms three H-bonds to the last T1 turn of the β-helix. In a trimer, three β-sandwiches are associated into a dome-like structure at the trimer interface (Fig. 2c). In addition, three pairs of Arg^555^ and Glu^596^, each from a different subunit, form three inter-chain salt bridges and three intra-chain H-bonds, constituting a triangular network that fastens the β-helix and β-sandwich domains together and firmly locks the three subunits (Additional file 1: Fig. S4c). This structure also seals off the central channel of the β-helix at the bottom.

### An unusual protruding structure stabilizes the central receptor-binding domain

While most T2 turns in the β-helix are restricted to two residues, the T1 and T3 turns vary significantly in length. In particular, T1 and T3 of rung 2 are the longest and are depicted above as the “rider” and “front feet” of a horse, respectively (Fig. 2e). The “rider” protrudes prominently from the border of the β-helix and exhibits a β-barrel-like fold composed of two three-stranded antiparallel β-sheets plus a short α-helix (Fig. 3c). The “rider” is mainly stabilized by hydrophobic interactions in the interior and four backbone H-bonds via its extended contacts with the T1 turns of rungs 6–8. As revealed by the DALI server, the “rider” is structurally homologous to the I-domain of bacteriophage P22 coat protein (a rms deviation of 3.20 Å between the Cα atoms) (Fig. 3d) [36]. This I-domain was reported to be crucial to the folding and assembly of the P22 capsid [37], suggesting that the “rider” in the K1 lyase has a role in stabilizing the structure of the enzyme. On the other hand, the “front feet” section is mainly made up of a six-residue 3_10_-helix (Fig. 2e). This motif is stabilized by interactions with adjacent T3 turns via a side-chain H-bond and van der Waals contacts.

In the trimer, three β-helices are packed laterally through the β-sheets b2 and b3, forming a parallel, left-handed superhelix with a triangular hydrophilic channel at the center. This superhelix is stabilized by 72 H-bonds and 18 salt bridges at the buried inter-subunit interfaces. Notably, the three “rider” domains further stabilize the superhelix (Fig. 2b–d), another uncommon feature in bacteriophage TSPs (Additional file 1: Fig. S3). The “rider” protrudes into the neighboring subunit and interacts with the “front feet” and its adjacent T3 turns (Fig. 3e). As a result, the three “rider” domains contribute 27 H-bonds and nine salt bridges, despite the modest buried interface (~ 1340 Å^2^). A virtually identical interaction was observed in the *C*2 crystal (Additional file 1: Fig. S2d). Mutations in the “rider” domain, such as the quadruple mutant K154A/K167A/T213A/H215A, significantly reduced the thermal stability of the enzyme (Fig. 3f), providing additional support for the structural importance of the part of the protein.

### The central receptor-binding domain contains two distinct carbohydrate-binding sites

The product complex structure at 1.46-Å resolution showed a K1 lyase trimer bound to five trisaccharides of [2,3-(*S*)-pyruvate]-β-D-∆4,5-Glc*p*A-(1 → 4)-*O*-acetyl-α-L-Fuc*p*-(1 → 3)-β-D-Glc*p*, each corresponding to one repeating unit of the K1 CPS [10]. Unexpectedly, the bound trisaccharides revealed two distinct carbohydrate-binding sites in the receptor-binding domain: the first in the solvent-accessible groove of each subunit, the second in an inter-subunit pocket beneath the “rider” (Fig. 4a). The bound trisaccharides did not induce significant conformational change of the protein (a rms deviation of ~ 0.22 Å from the free form structure between all Cα atoms), reflecting a rigid nature of the β-helix.

In the first binding site, the trisaccharide spans rungs 3–7 of the β-helix with main interactions to the protruding loops at T1 and T3 (Fig. 2d). It has an orientation nearly parallel to the longitudinal axis of the β-helix with the non-reducing end pointing to the N-terminus (Fig. 4a). Electron densities for the pyruvylation and the planar C-4–C-5 double bond of glucuronic acid as well as the acetylation at C-3 hydroxyl group of the fucose are clear (Fig. 4b, Additional file 1: Fig. S5). Altogether, the trisaccharide binding to the enzyme is mediated by 13 direct H-bonds: three between the glucuronic acid and amino acid residues Gln^130^ and Lys^291^; three between the fucose and Lys^291^, Glu^314^ and Asn^339^; and seven between the glucose and Tyr^186^, Glu^285^, Trp^310^, Tyr^311^, Asn^339^, Trp^340^ and His^373^ (Fig. 4c). In addition, there are four water-mediated H-bonds between the glucuronic acid and Gln^130^, Thr^255^, Asn^261^ and Asn^293^. The trisaccharide lies in a ~ 70-Å-long positively charged groove (Fig. 4a). The pyruvyl group of glucuronic acid fits well into a pocket lined by Ile^256^, Gly^286^ and Arg^287^ (Fig. 4d). Notably, the pyruvyl and acetyl groups of the trisaccharide are both involved in H-bonding to the enzyme.

In the second binding site, the unstructured loops of the “rider” domain, the adjacent T1 and B2 of rungs 7–10 and, from a neighboring β-helix, B3 and T3 of rungs 8–11, constitute the carbohydrate-binding pocket (Figs. 2d, 4a). The bound trisaccharide spans two adjacent β-helices and adopts an orientation nearly perpendicular to the longitudinal axis of the structure. The non-reducing end points to the central channel, with the reducing end oriented outward. The trisaccharide is stabilized by seven direct H-bonds: three between the glucuronic acid and Arg^472^/Arg^378^* (from another subunit); two between the fucose and Asp^434^; and two between the glucose and Lys^177^*/Asn^217^* (Fig. 4e). Moreover, there are five water-mediated H-bonds: four between the glucuronic acid and Tyr^409^/Arg^472^/Arg^475^/Lys^377^* and one between the fucose and Lys^377^*. The pyruvyl group of the glucuronic acid is docked into a positively charged pocket formed by Arg^472^/Thr^474^/Arg^475^ with five H-bonds, and its methyl group makes a further CH/π interaction with the imidazole ring of His^455^ (Fig. 4e, f). These findings suggest that the pyruvyl group serves as a determinant for recognition by the enzyme.

It is worth noting that the bound trisaccharides adopt distinct conformations between the catalytic and non-catalytic binding sites (Fig. 4b, g, Additional file 1: Fig. S5), as evident from the strikingly different dihedral angles, i.e., Ф and ψ [38], in the glycosidic linkage of β-Glc*p*A-(1 → 4)-Fuc*p* (Additional file 1: Table S1). This may suggest that the K1 CPS is flexible. Given the rigid architecture of the enzyme, a flexible substrate would presumably facilitate binding to both binding sites as well as the exposure of its pyruvyl group for recognition by the protein.

### Tyr^311^, His^373^ and Arg^397^ are important in catalysis

**Fig. 5:**
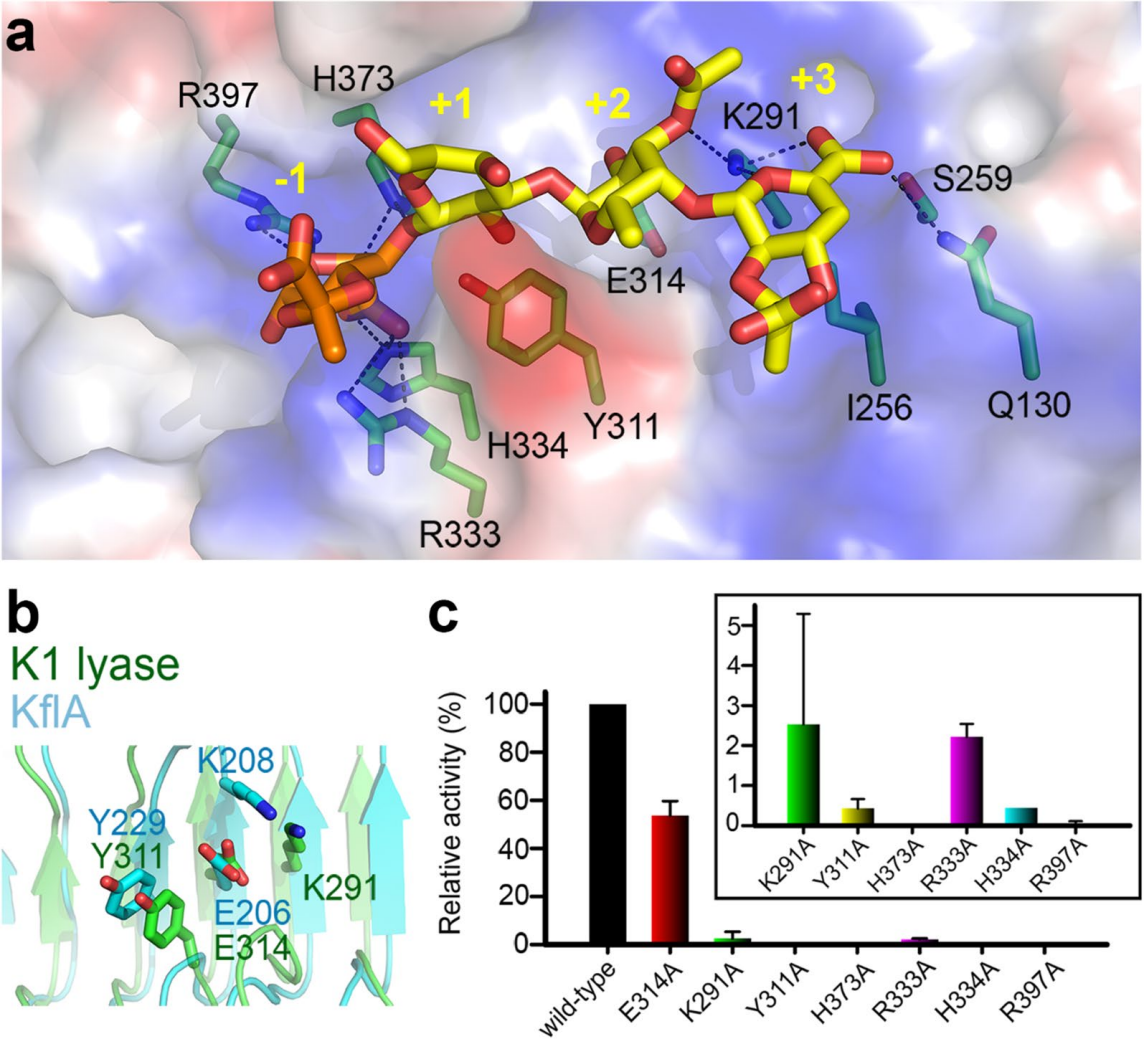
The catalytic site.** a** The residues (green) surrounding the bound trisaccharide (yellow) at the solvent-accessible groove of an individual subunit. A pyruvylated glucuronic acid (orange) is modeled to the reducing end of the bound trisaccharide as the − 1 site. **b** Superimposition of the catalytic center of the K1 lyase (green) with that of KflA (cyan), a bacteriophage-derived K5 lyase^22^. **c** Site-directed mutagenesis analyses of selected candidate residues that are proposed to be involved enzyme catalysis

Polysaccharide lyases catalyze the cleavage of uronic acid-containing polysaccharide chains via a β-elimination reaction to generate unsaturated oligosaccharide products (Additional file 1: Fig. S6) [34]. Published structures of polysaccharide lyases [34], including two derived from bacteriophage tailspikes [39, 40], have suggested residues serving as a Brønsted base/acid or neutralizer of the uronic carboxyl group. In the present study, examination of residues surrounding the bound products suggests that the catalytic site is adjacent to the reducing end of the trisaccharide in the solvent-accessible groove (Fig. 5a). Indeed, this area can be overlaid well with the proposed catalytic site of KflA, a bacteriophage-derived K5 lyase that is also a trimeric β-helical protein (Fig. 5b) [39]. Based on this finding, several possible catalytic residues such as Lys^291^, Glu^314^, Tyr^311^ and His^373^ were individually mutated to alanine (Fig. 5a). We found that the mutation H373A completely abolished enzymatic activity, while Y311A retained only ~ 0.4% activity (Fig. 5c). This is consistent with the structural observation that the side chains of Tyr^311^ and His^373^ are in proximity to the anomeric oxygen of glucose (Fig. 5a), thereby adjacent to the glucuronic acid where the β-elimination reaction occurs (Additional file 1: Fig. S6).

To identify the catalytic neutralizer of the uronic acid, we modeled a pyruvylated glucuronic acid to the reducing end as the − 1 site and found candidate residues such as Arg^333^, His^334^ and Arg^397^ (Fig. 5a). Indeed, in the *C*2 crystal form, a citrate molecule was bound to this − 1 site with its carboxyl groups forming H-bonds to all three candidate residues (Additional file 1: Fig. S2e). Hence, these amino acids were mutated to alanine individually. We found that the mutant R397A retained only ~ 0.04% enzymatic activity (Fig. 5c). Because treatment of the enzyme with EDTA showed little effect on the enzyme activity (data not shown), and no metal ion could be identified in the present structures, we deduce that Arg^397^ serves as a neutralizer for the carboxyl group. Indeed, the side chains of Tyr^311^ and His^373^ are also in close proximity to the C-4 and C-5 atoms of the modeled glucuronic acid.

### Arg^472^ in the second binding site, but not Arg^378^, is crucial to the function of K1 lyase and the absorption and infectivity of phage

**Fig. 6:**
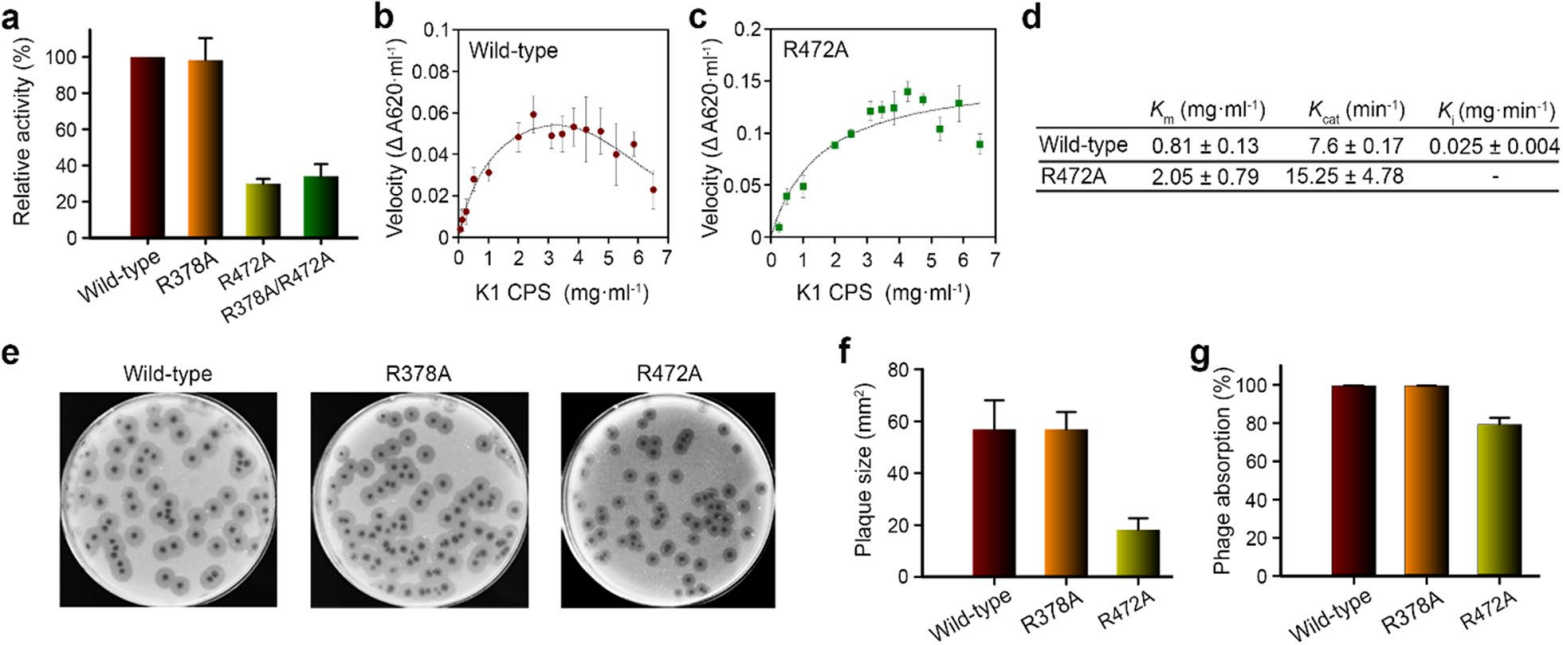
Inter-subunit carbohydrate-binding site is crucial to CPS depolymerization, phage absorption and infectivity. **a** Comparison of CPS depolymerization activities of wild-type and mutant K1 lyases evaluated by monitoring the increase of absorbance at 232 nm. **b** and **c** Enzyme kinetics study of wild-type K1 lyase and its R472A mutant, respectively. The results of initial velocity are shown as mean ± SD from triplicate experiments. **d** Kinetic parameters calculated from (**b**) and (**c**). **e** Plaque morphology of wild-type and mutant phages. The NTUH-K2044-K1-1 phage, with or without the mutations at the coding region of the K1 lyase, were spotted on plates pre-inoculated with K1 *K. pneumonia* NTUH-K2044. **f** Comparison of plaque sizes of wild-type and mutant phages as evaluated by measuring the diameters of 20 randomly selected plaques. Plaques from the phage with the R472A mutation in the K1 lyase were significantly reduced in size compared to wild type (*P* < 0.0001, Student’s t-test), whereas the phage with the R378A mutation showed little difference (*P* = 0.8703). **g** Comparison of absorption efficiencies of wild-type and mutant phages. The phage with the R472A mutation showed decreased absorption on NTUH-K2044 compared to wild type (*P* = 0.0013), but the phage with the R378A mutation had nearly no difference (*P* = 0.1012)

To probe the role of the second inter-subunit pocket for polysaccharide binding Arg^472^ and Arg^378^ were mutated to alanine. These residues are involved in interactions with the pyruvyl moiety (Arg^472^) and the carboxyl group (Arg^378^) of the glucuronic acid of the bound trisaccharide. Mutations R472A and R472A/R378A reduced the enzyme activity to ~ 30% and ~ 34%, respectively, but R378A had nearly no effect (Fig. 6a), suggesting that the pyruvyl group of glucuronic acid, rather than the carboxyl group, might serve as a recognition determinant for the enzyme, in agreement with the structural observations described above.

An enzyme kinetics study revealed possible product inhibition during catalysis, with a *K*_i_ value of 0.025 ± 0.004 mg min^−1^ (Fig. 6b, c). To explore if the bound trisaccharide at the second binding site interferes with substrate binding, we analyzed the kinetics of the R472A mutant and found that product inhibition became negligible in spite of a weaker binding affinity (~ 2.5-fold higher *K*_m_ value) of the mutant to the substrate (Fig. 6c, d). However, the mutant exhibited a two-fold higher turnover rate compared to that of the wild-type enzyme.

To evaluate the role of the second carbohydrate-binding site in phage infectivity, we generated two mutant phages that separately bear R378A and R472A mutations in the lyase. Remarkably, the R472A mutation reduced the plaque size of phage. On the other hand, the R378A mutation had nearly no effect (Fig. 6e, f). We also employed these mutant phages to evaluate the role of this site on phage attachment to *K. pneumoniae*. Absorption efficiency was attenuated by ~ 20% in the R472A mutant whereas the R378A mutant had nearly no effect (Fig. 6g).

### Administration of the K1 lyase, but not an inactive mutant, significantly increases survival rate in mice pre-infected with high-doses of *K. pneumonia*

**Fig. 7:**
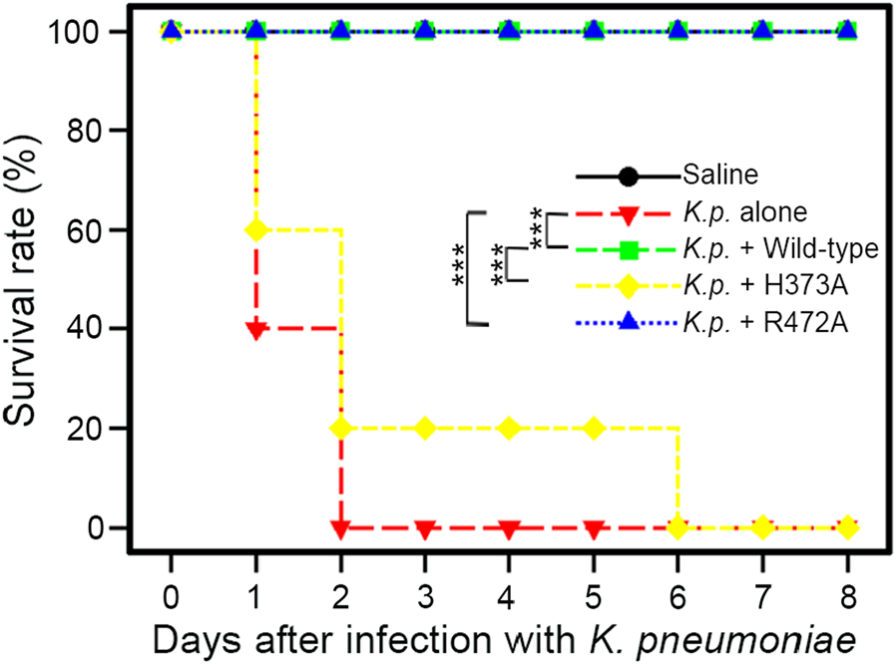
Efficacies of the K1 lyase and its mutants in protecting mice from high-dose *K. pneumoniae* infection. Three groups of mice, each containing five 5-week-old female BALB/cByl mice, were inoculated intraperitoneally with 8.0 × 10^7^ CFUs of NTHU-K2044. After 30 min, the mice were administrated intraperitoneally with 25 µg of the wild-type or mutant K1 lyase, as indicated. The other two groups of mice were inoculated with NTHU-K2044 alone or phosphate-buffered saline as the negative and positive control, respectively. ***P < 0.005, due to log-rank test

A previous study showed that treatment with recombinant K1 lyase promoted survival in mice pre-infected with NTHU-K2044 at a dose (3.3 × 10^3^ CFUs) close to the LD_50_ [15]. In the present study, mice were infected with a higher dose (8.0 × 10^7^ CFUs) of NTHU-K2044 to further evaluate the protection efficacy of the enzyme. Intraperitoneal infection with NTHU-K2044 resulted in 100% death (5/5) of the mice at day two, whereas all of the mice treated intraperitoneally with the K1 lyase (30 min after infection) survived through day eight (*P* = 0.0027, log-rank test, Fig. 7). Similarly, mice treated with the R472A mutant phage also completely survived at day eight (*P* = 0.0027), although the lyase has only ~ 30% activity compared to wild-type. In contrast, treatment with the inactive H373A mutant lyase resulted in 80% death (4/5) at day two and 100% death at day six. There was no significant change in the behavior or body weight of the mice (Additional file 1: Fig. S7), indicating its low toxicity [41].

## Discussion

Rapidly-developing molecular biology techniques and genetic tools are engendering novel and safe antimicrobial agents based on natural bacterial parasites, e.g., phages. Bacteriophage NTUH-K2044-K1-1 belongs to a new genus “*Kp34likevirus*” within the *Podoviridae* family [42]. Phages in this family share similar genome organization and conserved amino acid sequences of encoded proteins but display distinct host specificities, mainly attributed to different polysaccharide depolymerases in their tail [42]. However, the binding sites and the catalytic machineries of phage-derived polysaccharide depolymerases remain difficult to predict from sequences without 3D-structural information.

We demonstrate here that the polysaccharide depolymerase from the bacteriophage NTUH-K2044-K1-1 is a lyase, that catalyzes a *syn*-β-elimination reaction using the CPS of NTUH-K2044 *K. pneumoniae* as a substrate. The crystal structure of the enzyme in complex with its product represents the first structure of a carbohydrate-bound bacteriophage-derived polysaccharide lyase. This work thus provides the first insights into how viral polysaccharide lyases recognize and depolymerize the CPS of their bacterial hosts. The structures not only exhibit typical features of bacteriophage TSPs, such as the central β-helix and the C-terminal β-sandwich domains, but also contain unusual features, such as an N-terminal collagen-like trimerization fold and a striking protruding domain from the β-helix (Additional file 1: Fig. S3). Interestingly, a triple-helix bundle that connects the N-terminal particle-binding domain and central receptor-binding domain is common in reported TSP structures but missing in the present structures (Additional file 1: Fig. S3) [39, 43, 44]. This counters the recently proposed role of the helix bundle in signal transmission from the distal tip of TSP to the phage head that eventually leads to DNA ejection [43].

Unlike other TSPs, such as the P22 TSP, KflA and ΦAB6 TSPs, which contain a β-prism or triple-stranded β-helix following the central β-helix [39, 43, 44], the K1 lyase lacks the interdigitated or swapped elements between subunits (Additional file 1: Fig. S3). The human collagen XVIII trimerization domain contains swapped loops between subunits, thus creating numerous interactions at the trimer interfaces [35], but no swapping was found in the N-terminal domain of the K1 lyase, despite the similar fold (Fig. 3b). In this regard, the buried interfaces in the present structures are probably more accessible to the solvent, accounting for the weaker tolerance of the enzyme to SDS denaturation. In contrast, the enzyme showed higher thermal stability compared to other TSPs, likely due to the additional stabilization contributed by the protruding “rider” domain, as supported by our mutagenesis study. It is intriguing to contemplate engineering the N-terminal domain to be even more collagen-like to further improve thermal stability.

The striking protruding domain at the central β-helix described here is uncommon in bacteriophage TSPs (Additional file 1: Fig. S3) [39, 43–46]. The P22 TSP structure also has a protruding domain on the central β-helix, displaying a shape like the dorsal fin of a fish [43]. However, unlike the “rider” of the K1 lyase, the “dorsal fin” of P22 TSP is located in a T3 turn and makes only a slight contact with the neighboring β-helix. This “dorsal fin” does not interact with the polysaccharide substrate [47]. In contrast, the “rider” of K1 lyase participates in polysaccharide binding to both carbohydrate-binding sites and makes an important contribution to the structural stability of the trimeric enzyme.

The first carbohydrate-binding site of the K1 lyase is in an elongated positively-charged groove that extends longitudinally for ~ 70 Å. The distance between the reducing end of the bound trisaccharide and the C-terminal end of the enzyme is more than 40 Å (Fig. 4a). Thus, this solvent-accessible groove is capable of accommodating oligosaccharides up to three repeating units, likely reflecting the maximum size of K1 CPS degradation products. Thirteen direct H-bonds stabilize the trisaccharide product at the groove with seven between the reducing-end glucose and the enzyme. Prediction of p*K*_a_ values for nearby ionizable residues with the PDB2PQR server (http://nbcr-222.ucsd.edu/pdb2pqr_2.1.1) indicates that the p*K*_a_ of Tyr^311^ shifts from 10.07 to 13.24, and His^373^ from 6.10 to 3.44, suggesting that Tyr^311^ and His^373^ serve as the Brønsted base and acid, respectively. Consequently, the above H-bond network probably forces the anomeric oxygen of glucose in the polysaccharide to be in the proximity of Tyr^311^ and His^373^, facilitating catalysis. In addition, according to its position near the carboxyl group of the modeled glucuronic acid, and supported by mutagenesis analysis, Arg^397^ probably serves as a neutralizer to lower the p*K*_a_ value of the C-5 proton of the glucuronic acid.

This study provides the first direct evidence to identify a second, non-catalytic, carbohydrate-binding site in bacteriophage TSPs. The bound trisaccharide at the inter-subunit binding site occupies most of the pocket, and its non-reducing end nearly reaches the bottom, suggesting that the pocket can only accommodate the terminal portion of the K1 CPS. Although the depolymerization reaction takes place in each individual subunit, the results from SDS dissociation and mutagenesis analysis indicate that the inter-subunit binding site plays an important role in CPS depolymerization. Furthermore, this inter-subunit binding pocket is also important for phage attachment and infection to *K. pneumoniae*, as supported by the results from phage absorption and infectivity studies. Notably, the pyruvyl group of the bound trisaccharide at the non-catalytic site is embedded deep in a pocket, reminiscent of the pseudaminic acid in the surface polysaccharide of the bacterium *Acinetobacter baumannii* [48]. This pseudaminic acid serves as a recognition site for ΦAB6 TSP, another bacteriophage-derived polysaccharide hydrolase [48]. In this regard, the pyruvyl group of K1 CPS might likewise serve as a recognition site for K1 lyase. Through a mutagenesis study, we demonstrated that the interaction between the pyruvyl group and the enzyme is crucial to CPS depolymerization and phage absorption and infection. Indeed, the pyruvyl group of K1 CPS has been reported to be an important recognition site for the host immune system [10, 49]. This implies that the pyruvylation site can be a target for designing vaccines and therapeutic antibodies.

Bacteriophage polysaccharide depolymerases that recognize specific exopolysaccharides of pathogenic bacteria can be advantageous in therapeutic use, such as selective killing of a pathogen without affecting normal microflora and reducing the development of antibiotic resistance [50]. In the present study, we demonstrate that the K1 lyase protects mice from K1 *K. pneumoniae* infection*,* even with a high challenge dose. Presumably, the enzyme removes the CPS from the bacteria and the loss of capsule enables the host killing by the immune system. The lack of adverse effects in the mice suggests low toxicity of the enzyme. We also demonstrated that the K1 lyase-digested CPS products contained pyruvylation and *O*-acetylation, thus retaining the important antigens of intact K1 CPS, as evident from the observation that the antiserum against intact K1 CPS strongly recognized the digested products. The finding that a single-residue mutation at the second carbohydrate-binding site of K1 lyase resulted in negligible product inhibition during catalysis might provide clues to protein engineering for optimizing the catalytic efficiency of the enzyme. These results imply the application of this enzyme as well as its digested products in preparation of therapeutic agents and glycoconjugate vaccines against PLA *K. pneumoniae* infection.

## Conclusions

We demonstrate here that the K1 lyase from the NTUH-K2044-K1-1 bacteriophage is a polysaccharide depolymerase that cleaves the CPS into oligosaccharides of 1–3 repeating trisaccharide units with the retention of the pyruvylation and *O*-acetylation. This enzyme has a trimeric β-helix architecture and contains two distinct carbohydrate-binding sites. One is responsible for CPS depolymerization via a *syn* β-elimination mechanism that involves Tyr^311^, His^373^ and Arg^397^. The other also recognizes and binds to CPS but is not catalytic, and a single-residue mutation, R472A in this site, liberated the enzyme from product inhibition during catalysis. The tight interaction between the pyruvate moiety of the CPS and the enzyme in the second binding site, mediated by Arg^472^, is also crucial to CPS depolymerization as well as phage absorption and infectivity. Administration of the enzyme, but not an inactive mutant, protected mice from infection by a high-dose of K1 *K. pneumonia* without significant adverse effects. Our results provide insights into how the enzyme recognizes and depolymerizes the CPS, and demonstrate the potential use of the protein not only as a therapeutic agent against *K. pneumoniae*, but also as a tool to prepare structurally-defined oligosaccharides for the generation of glycoconjugate vaccines against infections caused by this organism.

## Supplementary Information

**Additional file 1: Figure S1. **Characterization of recombinant K1 lyase. **Figure S2**. Crystal structure of K1 lyase in the C2 crystal form. **Figure S3.** Comparison of the K1 lyase structure described here with some reported structures of bacteriophage-derived CPS depolymerases. **Figure S4.** The inward and outward side-chain stacks and the triangular interaction network at the central β-helix of K1 lyase. **Figure S5.** The 2Fo-Fc omit maps for the bound trisaccharides [2,3-(S)-pyruvate]-β-D-∆4,5-GlcpA-(1→4)-O-acetyl-α-L-Fucp-(1→3)-β-D-Glcp at the first (or catalytic) and second (or non-catalytic) binding sites. **Figure S6.** Schematic illustration of the sym β-elimination reaction catalyzed by K1 lyase. **Figure S7.** Effect of high-dose K1 lyase on the survival rate and body weight of mice. **Table S1.** The dihedral angles (Ф, ψ) of glycosidic linkages of the bound trisaccharides at the catalytic and non-catalytic binding sites of K1 lyase.

## Data Availability

The atomic coordinates and structure factors of the crystal structures in this study have been deposited in the Protein Data Bank (www.wwpdb.org) under accession codes 7W1C, 7W1D and 7W1E. Other data and materials are available from the corresponding authors on reasonable request.

## Abbreviations

CPS: Capsular polysaccharide
PLA: Pyogenic liver abscess
TSP: Tailspike protein
