# Supplementary material for: Structural and biological insights into Klebsiella pneumoniae surface polysaccharide degradation by a bacteriophage K1 lyase: implications for clinical use
Source: J Biomed Sci. 2022 Feb 7;29:9. doi: 10.1186/s12929-022-00792-4 (PMC8822698; doi:10.1186/s12929-022-00792-4)
Supplement: Supplementary file 1 — Additional file 1: Figure S1. Characterization of recombinant K1 lyase. Figure S2. Crystal structure of K1 lyase in the C2 crystal form. Figure S3. Comparison of the K1 lyase structure described here with some reported structures of bacteriophage-derived CPS depolymerases. Figure S4. The inward and outward side-chain stacks and the triangular interaction network at the central β-helix of K1 lyase. Figure S5. The 2Fo-Fc omit maps for the bound trisaccharides [2,3-(S)-pyruvate]-β-D-∆4,5-GlcpA-(1→4)-O-acetyl-α-L-Fucp-(1→3)-β-D-Glcp at the first (or catalytic) and second (or non-catalytic) binding sites. Figure S6. Schematic illustration of the sym β-elimination reaction catalyzed by K1 lyase. Figure S7. Effect of high-dose K1 lyase on the survival rate and body weight of mice. Table S1. The dihedral angles (Ф, ψ) of glycosidic linkages of the bound trisaccharides at the catalytic and non-catalytic binding sites of K1 lyase. [file 12929_2022_792_MOESM1_ESM.pdf]

Supplementary Information

Structural and biological insights into *Klebsiella pneumoniae* surface polysaccharide degradation by a bacteriophage K1 lyase: implications for clinical use

I-Fan Tu[1†], Tzu-Lung Lin[2†], Feng-Ling Yang[1], I-Ming Lee[1], Wei-Lin Tu[1], Jiahn-Haur Liao[1], Tzu-Ping Ko[1], Wen-Jin Wu[1], Jia-Tsrong Jan[3], Meng-Ru Ho[1], Ching-Yi Chou[1], Andrew H.-J. Wang[1], Chung-Yi Wu[3], Jin-Town Wang[2], Kai-Fa Huang[1]* and Shih-Hsiung Wu[1,4]*

[1]Institute of Biological Chemistry, Academia Sinica, Taipei 115, Taiwan
[2]Department of Microbiology, National Taiwan University Hospital, Taipei 100, Taiwan
[3]Genomics research center, Academia Sinica, Taipei 115, Taiwan
[4]Department of Chemistry, National Taiwan University, Taipei 106, Taiwan

*Correspondence: huangkf@gate.sinica.edu.tw; shwu@gate.sinica.edu.tw
[1]Institute of Biological Chemistry, Academia Sinica, No. 128 Academia Road Section 2, Nan-Kang, Taipei 115, Taiwan

[†]I-Fan Tu and Tzu-Lung Lin contributed equally to this work.

The file includes:

Figures S1 to S7
Table S1

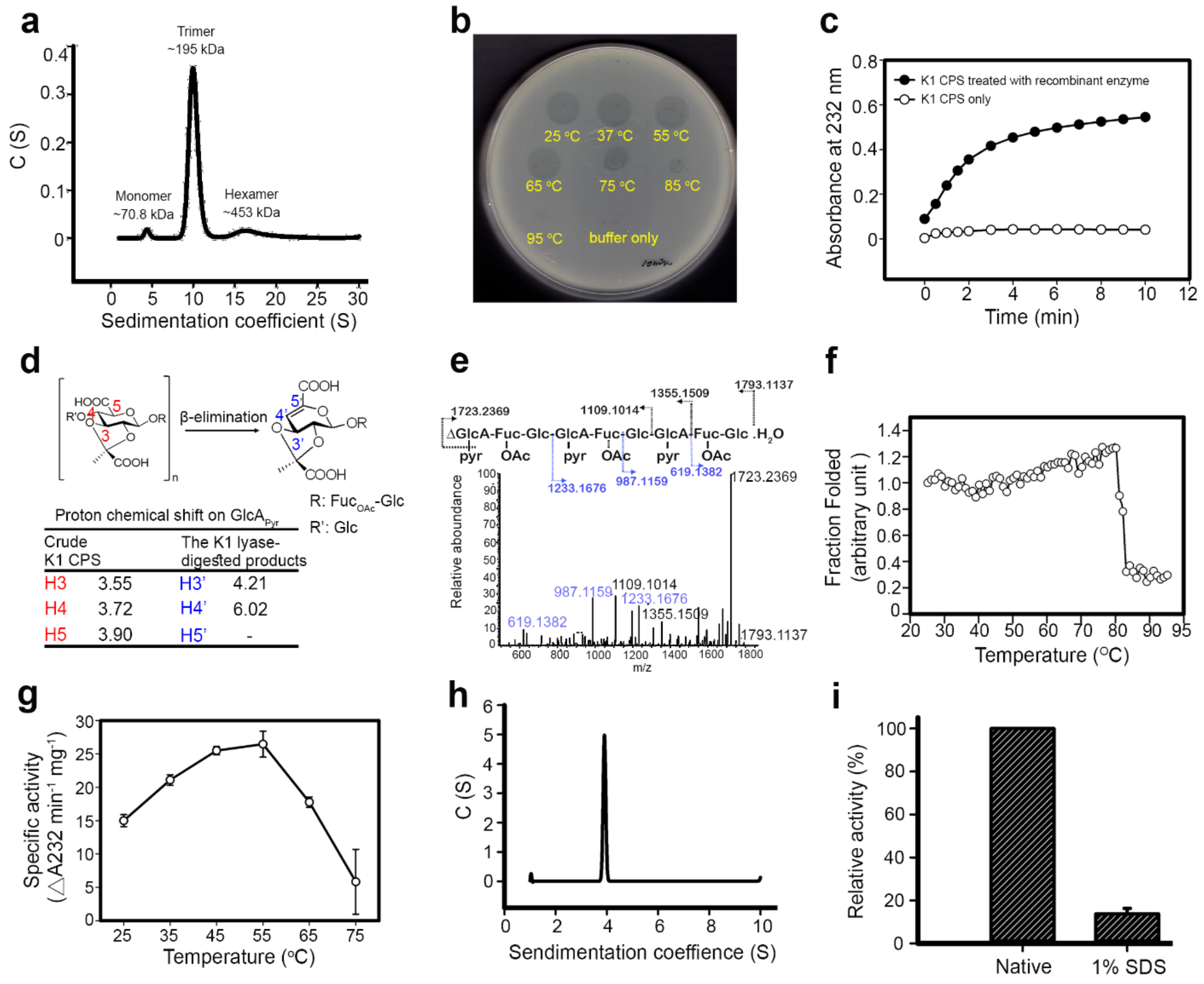

Fig. S1. Characterization of recombinant K1 lyase. **a** Assembly of K1 lyase in solution as determined by analytical ultracentrifugation. **b** Top agar assay of K1 lyase after heating at varying temperatures, as indicated, for 5 min. **c** Enzyme activity assay of K1 lyasae on the substrate K1 CPS as evaluated by monitoring the elevated UV absorption at 232 nm. **d** Proton chemical shifts on GlcA of K1 CPS after treatment with K1 lyase, as measured by 2D $^{1}H,^{1}H$ DQF-COSY NMR spectrum. **e** LC-ESI-MS-MS analysis of a K1 lyase-digested product (m/z = 1811.46) of K1 CPS. **f** Thermal stability of K1 lyase as evaluated by circular dichroism spectroscopy. **g** Enzymatic activity of K1 lyase under varying temperatures. The results are shown as mean ± SD from triplicate experiments. **h** Dissociation of trimeric K1 lyase into monomers in the presence of 1% SDS as analyzed by analytical ultracentrifugation. **i** Enzymatic activity of K1 lyase in the presence or absence of 1% SDS.

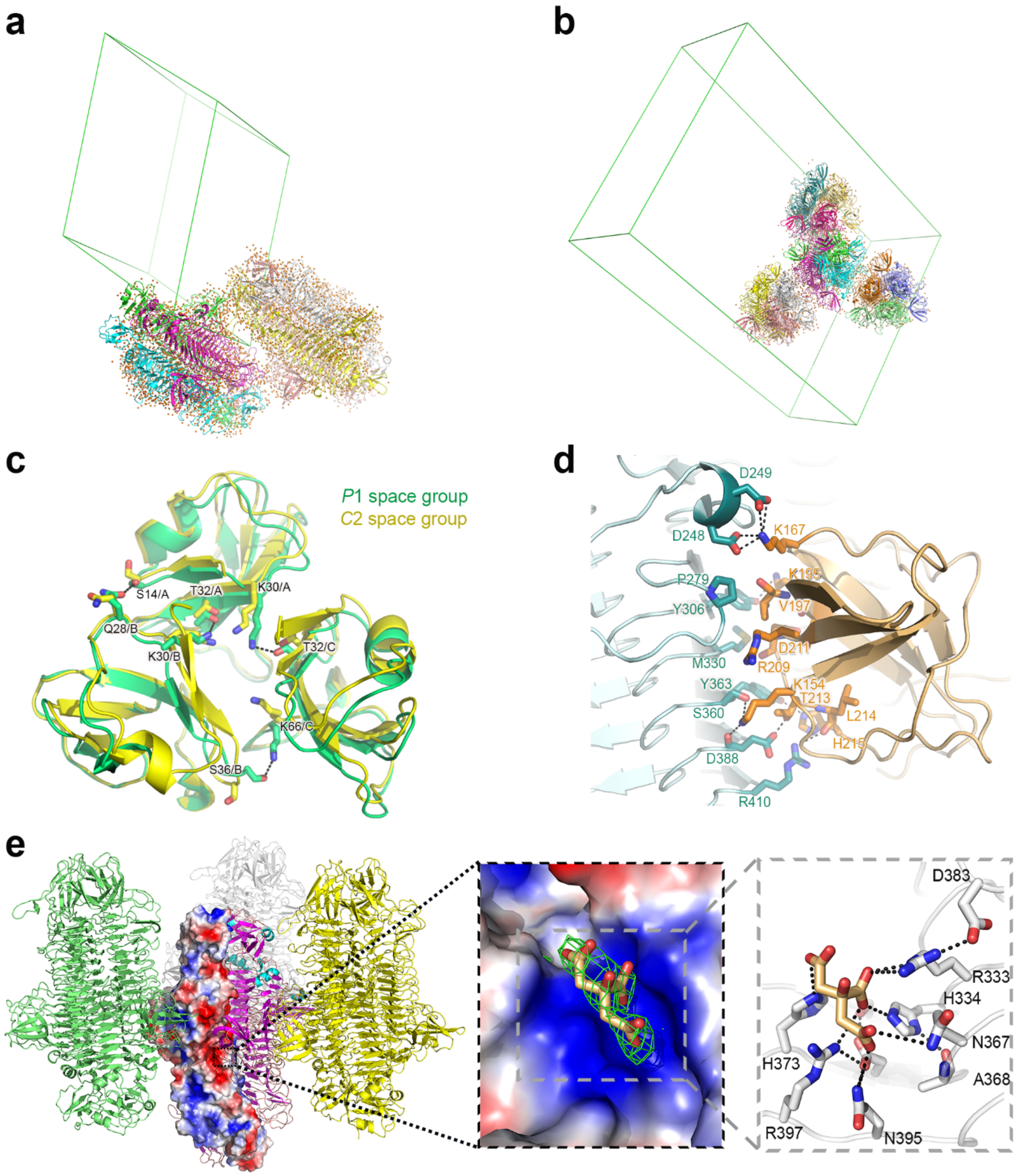

Fig. S2. Crystal structure of K1 lyase in the *C*2 crystal form. **a** Two K1 lyase trimers in the asymmetric unit of *P1* crystal form. The orange dots represent the solvent molecules. **b** Four K1 lyase trimers in the asymmetric unit of *C*2 crystal form. **c** Superimposition of K1 lyase trimers from the two crystal forms. The view from the N-terminal domains with the distinct hydrogen bonds between the two structures being indicated. **d** Interaction of the "rider" domain (orange) with the β-helix of a neighboring subunit (cyan) in the *C*2 crystal form. **e** A citrate molecule bound to the catalytic carbohydrate-binding site of K1 lyase in the *C*2 crystal form. The detailed interaction and 1σ F_o-F_c omit map for the bound citrate are shown.

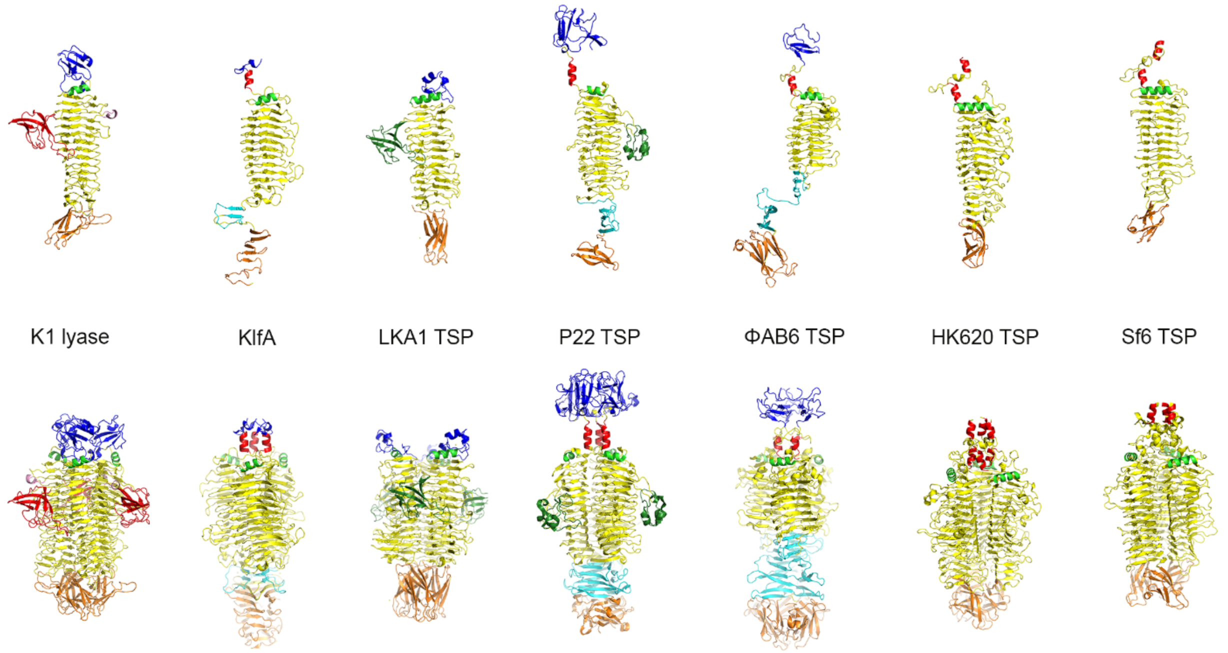

Fig. S3. Comparison of the K1 lyase structure described here with some reported structures of bacteriophage-derived CPS depolymerases. KflA: K5 lyase A[1]. LKA1 TSP: the phage LKA1 TSP with a polysaccharide lyase activity[2]. P22 TSP, ΦAB6 TSP, HK620 TSP and Sf6 TSP: the phage TSP with polysaccharide hydrolase activity from the bacteriophage P22[3], ΦAB6[4], HK620[5], and Sf6[6], respectively. Upper panel: the monomer. Lower panel: the trimer. The structure colored in blue, yellow and orange represent the N-terminal, β-helix and C-terminal domain, respectively. The helix bundle connecting the N-terminal and β-helix domains is colored red. The "cap" α-helix on the top of β-helix domain is colored green. The strikingly protruding structure at the β-helix of K1 lyase and other TSPs is colored deep red and deep green, respectively. The interdigitated or swapped structures between the β-helix and C-terminal domain are colored cyan.

References

1. Thompson, J.E.; Pourhossein, M.; Waterhouse, A.; Hudson, T.; Goldrick, M.; Derrick, J.P.; Roberts, I.S. The K5 lyase KflA combines a viral tail spike structure with a bacterial polysaccharide lyase mechanism. *J. Biol. Chem.* **2010**, *285*, 23963-23969.
2. Olszak T.; Shneider M.M.; Latka A.; Maciejewska B.; Browning C.; Sycheva L.V.; Cornelissen A.; Danis-Wlodarczyk K.; Senchenkova S.N.; Shashkov A.S.; et al. The O-specific polysaccharide lyase from the phage LKA1 tailspike reduces Pseudomonas virulence. *Sci. Rep.* **2017**, *7*, 16302.
3. Seul, A.; Müller, J.J.; Andres, D.; Stettner, E.; Heinemann, U.; Seckler, R. Bacteriophage P22 tailspike: structure of the complete protein and function of the interdomain linker. *Acta Crystallogr. D Biol. Crystallogr.* **2014**, *70*, 1336-1345.
4. Lee, I.M.; Tu, I.F.; Yang, F.L.; Ko, T.P.; Liao, J.H.; Lin N.T.; Wu, C.Y.; Ren, C.T.; Wang,

A.H.; Chang, C.M.; et al. Structural basis for fragmenting the exopolysaccharide of *Acinetobacter baumannii* by bacteriophage PhiAB6 tailspike protein. *Sci. Rep.* **2017**, *7*, 42711.

5. Barbirz S.; Muller J.J.; Uetrecht C.; Clark A.J.; Heinemann U.; Seckler R.; Crystal structure of Escherichia coli phage HK620 tailspike: podoviral tailspike endoglycosidase modules are evolutionarily related. *Mol. Microbiol.* **2008**, *69*, 303-316.
6. Muller J.J.; Barbirz S.; Heinle K.; Freiberg A.; Seckler R.; Heinemann U.; An intersubunit active site between supercoiled parallel beta helices in the trimeric tailspike endorhamnosidase of Shigella flexneri Phage Sf6. *Structure.* **2008**, *16*, 766-775.

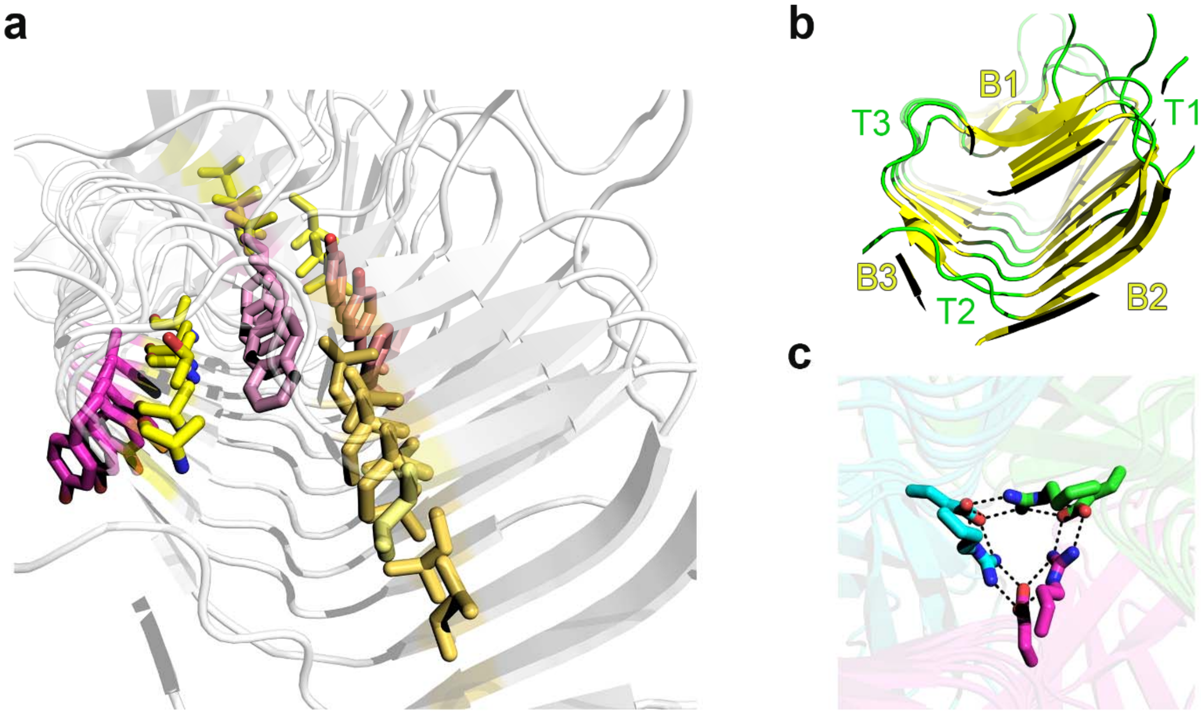

Fig. S4. The inward and outward side-chain stacks and the triangular interaction network at the central β-helix of K1 lyase. **a** The inward-facing β-branched stacks of Val, Ile, Leu and Met residues are colored yellow. The aromatic stacks of Phe and Tyr residues are colored purple. The outward-oriented stacks of Tyr and His residues are colored magenta. **b** The central β-helix is collapsed into a kidney-shaped cross section. Individual rung of the β-helix is organized into three strands, B1, B2 and B3, separated by turns T1, T2 and T3. **c** Three pairs of Arg^{555} and Glu^{596}, each from a different subunit, form three inter-chain salt bridges and three intra-chain hydrogen bonds, which constitute the triangular interaction network near the C-terminal end of β-helix.

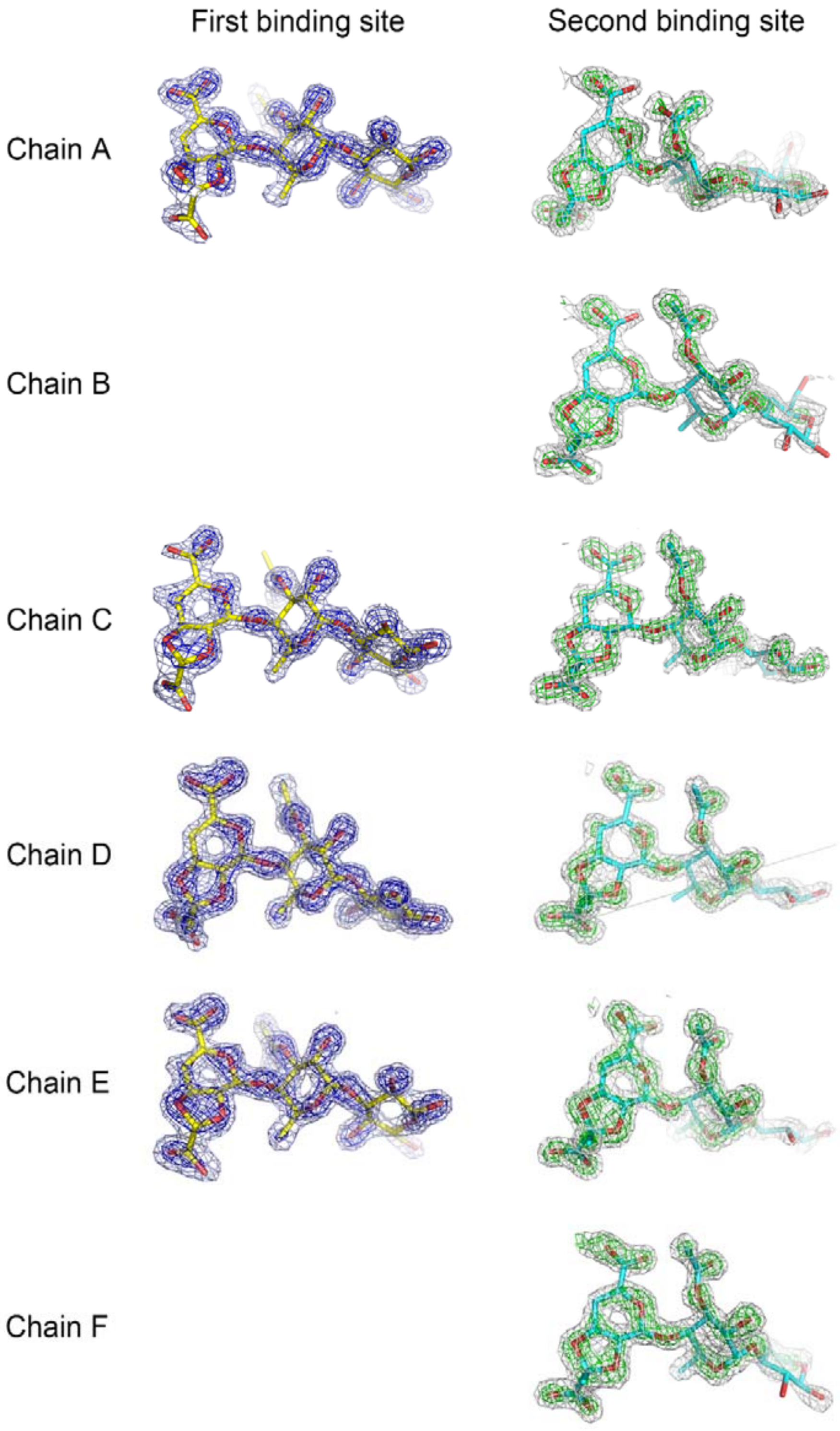

Fig. S5. The $2F_o$-F_c omit maps for the bound trisaccharides [2,3-(*S*)-pyruvate]-β-D-Δ4,5-Glc*p*A-(1→4)-*O*-acetyl-α-L-Fuc*p*-(1→3)-β-D-Glc*p* at the first (or catalytic) and second (or non-catalytic) binding sites. The stick models of the trisaccharides at the first and second binding site are colored yellow and cyan, respectively. The electron density maps contoured at 2.0σ are colored gray and 3.0σ colored blue or green.

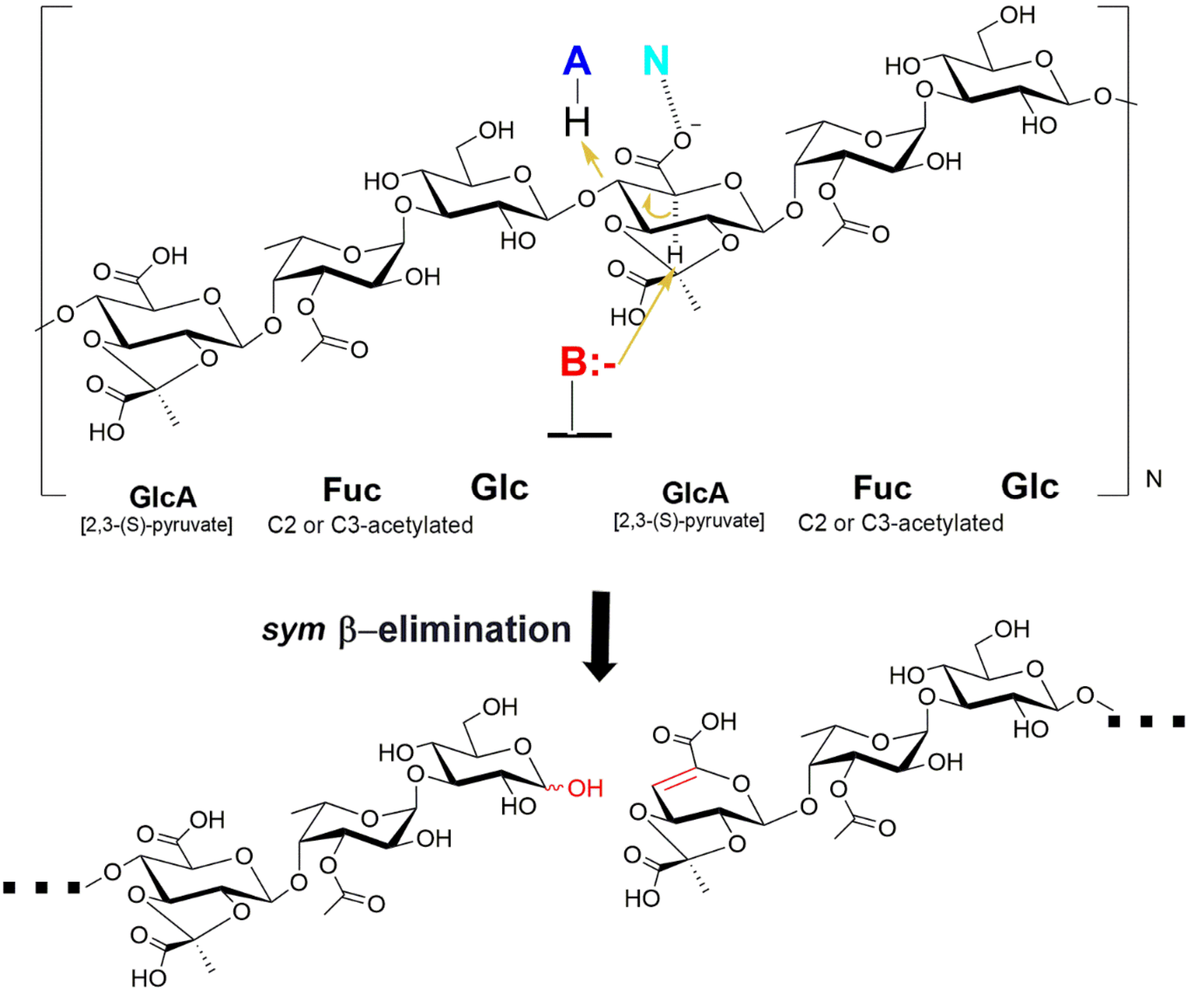

Fig. S6. Schematic illustration of the *sym* β-elimination reaction catalyzed by K1 lyase. Abbreviations: B, the Brønsted base; A, the Brønsted acid; N, the neutralizer.

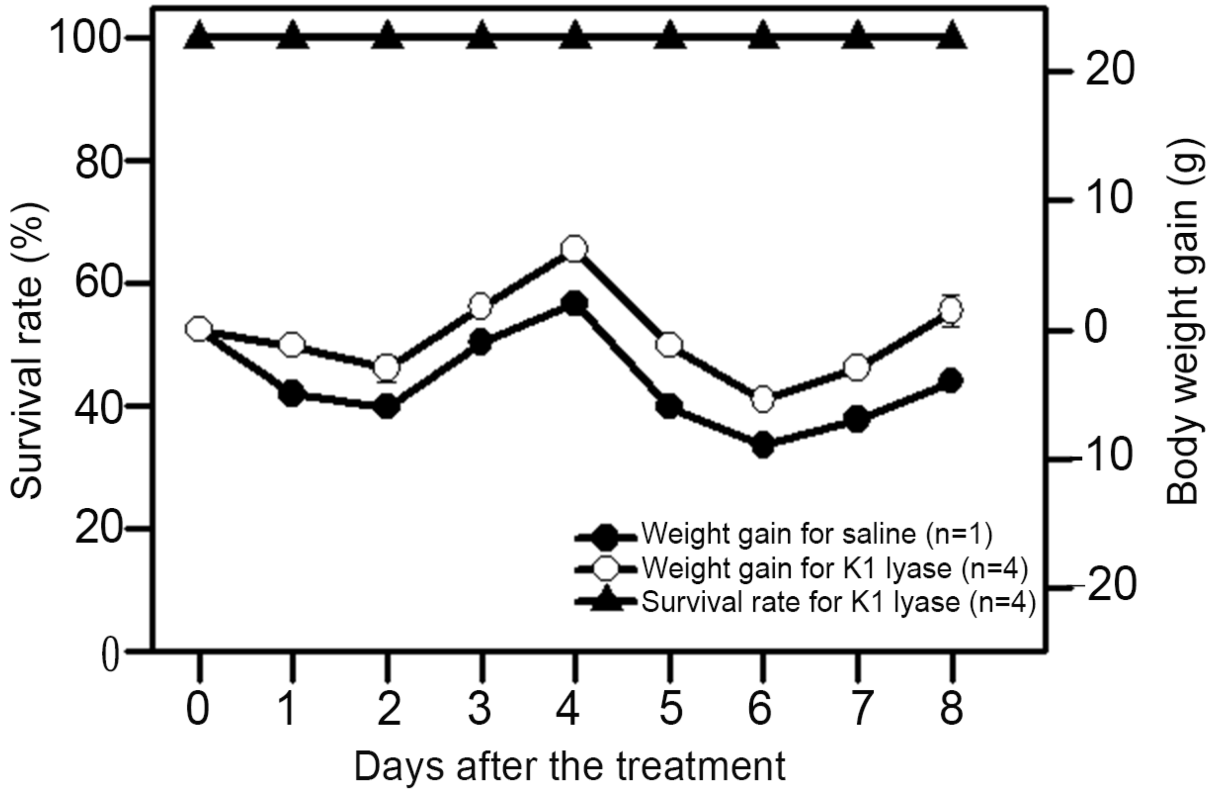

Fig. S7. Effect of high-dose K1 lyase on the survival rate and body weight of mice. Four 5-week-old female BALB/cByl mice were administrated intraperitoneally with K1 lyase at the dose of 100 μg per mouse and the survival rate and body weight gain were monitored for 8 days. A mouse injected with phosphate-buffered saline served as the control for body weight. The results of the K1 lyase treatments are shown as average ± SD.

Table S1. The dihedral angles (Φ, ψ) of glycosidic linkages of the bound trisaccharides at the catalytic and non-catalytic binding sites of K1 lyase

Glycosidic linkage	**GlcA-(β1→4)-Fuc**		**Fuc-(α1→3)-Glc**	
	Φ	ψ	Φ	ψ
Catalytic site	-97.6 ± 2.7	115.6 ± 0.7	-70.8 ± 0.7	-92.0 ± 1.0
Non-catalytic site	-58.0 ± 2.1	127.3 ± 0.6	-74.0 ± 8.4	-92.7 ± 9.7
